# Probing the accuracy and precision of Hirshfeld atom refinement with *HARt* interfaced with *Olex2*

**DOI:** 10.1107/S2052252517015548

**Published:** 2018-01-01

**Authors:** Malte Fugel, Dylan Jayatilaka, Emanuel Hupf, Jacob Overgaard, Venkatesha R. Hathwar, Piero Macchi, Michael J. Turner, Judith A. K. Howard, Oleg V. Dolomanov, Horst Puschmann, Bo B. Iversen, Hans-Beat Bürgi, Simon Grabowsky

**Affiliations:** a Department 2: Biology/Chemistry, University of Bremen, Leobener Straße NW2, 28359 Bremen, Germany; b School of Chemistry and Biochemistry, University of Western Australia, 35 Stirling Highway, Perth, WA 6009, Australia; c Center for Materials Crystallography, Department of Chemistry and iNano, Aarhus University, Langelandsgade 140, Aarhus 8000, Denmark; d Division of Physics, Faculty of Pure and Applied Sciences, University of Tsukuba, Tsukuba 305-8571, Japan; e Department of Chemistry and Biochemistry, University of Bern, Freiestraße 3, Bern 3012, Switzerland; f Department of Chemistry, Durham University, South Road, Durham DH1 3LE, UK; g Department of Chemistry, University of Zürich, Winterthurerstraße 190, Zürich 8057, Switzerland

**Keywords:** Hirshfeld atom refinement, multipole modelling, anisotropic displacement parameters, hydrogen-atom properties, crystallographic software

## Abstract

Hirshfeld atom refinement (HAR) is a novel X-ray structure refinement technique that employs aspherical atomic scattering factors obtained from stockholder partitioning of a theoretically determined tailor-made static electron density. HAR overcomes many of the known limitations of independent atom modelling (IAM), such as too short element–hydrogen distances, *r*(*X*—H), or too large atomic displacement parameters (ADPs). This study probes the accuracy and precision of anisotropic hydrogen and non-hydrogen ADPs and of *r*(*X*—H) values obtained from HAR. These quantities are compared and found to agree with those obtained from (i) accurate neutron diffraction data measured at the same temperatures as the X-ray data and (ii) multipole modelling (MM), an established alternative method for interpreting X-ray diffraction data with the help of aspherical atomic scattering factors. Results are presented for three chemically different systems: the aromatic hydro­carbon rubrene (orthorhombic 5,6,11,12-tetra­phenyl­tetracene), a co-crystal of zwitterionic betaine, imidazolium cations and picrate anions (BIPa), and the salt potassium hydrogen oxalate (KHOx). The non-hydrogen HAR-ADPs are as accurate and precise as the MM-ADPs. Both show excellent agreement with the neutron-based values and are superior to IAM-ADPs. The anisotropic hydrogen HAR-ADPs show a somewhat larger deviation from neutron-based values than the hydrogen SHADE-ADPs used in MM. Element–hydrogen bond lengths from HAR are in excellent agreement with those obtained from neutron diffraction experiments, although they are somewhat less precise. The residual density contour maps after HAR show fewer features than those after MM. Calculating the static electron density with the def2-TZVP basis set instead of the simpler def2-SVP one does not improve the refinement results significantly. All HARs were performed within the recently introduced *HARt* option implemented in the *Olex2* program. They are easily launched inside its graphical user interface following a conventional IAM.

## Abstract 2

Anisotropic atomic displacement parameters obtained separately from highly accurate X-ray and neutron diffraction data are compared, and it is established that Hirshfeld atom refinement of X-ray data can provide structural parameters that are as accurate as those from neutron data.

## 1. Introduction   

X-ray diffraction experiments provide access to the thermally smeared electron-density distribution, which is generally approximated as a convolution of a static electron density with a probability density function for the motion of the nuclei (Stewart & Feil, 1980 ▸). The static electron density of the unit cell is usually represented by a sum of atom-centred densities. In the simplest approximation these densities are assumed to be spherical free-atom densities. This approximation, the so-called independent atom model (IAM), has been used in hundreds of thousands of crystal structure refinements. However, if the effects of chemical bonding are taken into account with non-spherical static atomic electron densities, the refined atomic positions and anisotropic displacement parameters (ADPs) may differ favourably from those obtained with the IAM. While the positions of non-hydrogen atoms are often within 0.01–0.02 Å of those from IAM X-ray refinements, the hydrogen-atom positions and ADPs show considerable discrepancies (Coppens, 1997 ▸). Hydrogen–element bond distances in IAMs are underestimated by about 0.1 Å because the single electron of the H atom has to account for both the density around the proton and that in the hydrogen–element bond. Such were some of the original motivations for introducing the so-called X–N refinements, where X-ray data are refined with hydrogen positions and ADPs fixed at the values obtained from neutron diffraction data (Coppens, 1967 ▸; Figgis *et al.*, 1993 ▸). Throughout the past four decades a range of elaborate multipolar atomic density models (MM) have been introduced to counterbalance asphericity shifts, to capture the finer aspherical details of the charge density and to account for the effects of chemical bonding (Stewart, 1969 ▸; Hirshfeld, 1971 ▸; Kurki-Suonio, 1968 ▸; Hansen & Coppens, 1978 ▸; Destro *et al.*, 1988 ▸; Gatti *et al.*, 2002 ▸). Nevertheless, MM refinement of the ADPs of hydrogen atoms is possible only in exceptional cases (Zhurov *et al.*, 2011 ▸); it is not normally considered a viable option in multipole refinements (Hoser *et al.*, 2009 ▸).

The aim of the X-ray charge-density field has always been to obtain as accurate a description of the static electron density as possible by deconvolving and removing the effect of the thermal motion of the atoms. Such static densities are then used to study the chemical bonding in crystals (Koritsanszky & Coppens, 2001 ▸). However, multipole parameters, ADP values or both are prone to a range of systematic errors in the X-ray diffraction data (extinction, absorption, thermal diffuse scattering, integration errors *etc.*; Iversen *et al.*, 1999 ▸). In addition, refined ADPs may be biased due to incomplete models of the electron density and of atomic motion (Coppens, 1997 ▸).

One way to test the accuracy of an X-ray charge-density model is to compare the refined atomic positions and ADPs with values obtained from independent neutron diffraction experiments at matching temperatures (Morgenroth *et al.*, 2008 ▸; Jørgensen *et al.*, 2014 ▸), and the refined electron density with the density calculated from high-level *ab initio* methods. While atomic positions from X-ray and neutron diffraction usually agree well, the corresponding ADPs require a more detailed assessment. Measures for the agreement include the mean ratio of the diagonal X-ray (X) and neutron (N) ADPs, 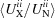, and the mean absolute difference of X-ray and neutron ADPs, 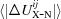. Another measure, the error-weighted root-mean-square difference (wRMSD) 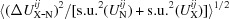, takes into account that neutron diffraction experiments are also prone to systematic and random errors (standard uncertainties, s.u.s) which cannot be unravelled with the statistical measures used. Thus, comparison of independent experiments in terms of their wRMSDs is the best available option. A comparison of experimental with calculated ADPs is not yet possible since accurate ADP values are not yet obtainable from *ab initio* theory (Madsen *et al.*, 2013 ▸).

Accurate static electron densities can be estimated routinely even in complex crystals. It may therefore be argued that unique scientific information can be retrieved from X-ray diffraction data if an accurate calculated static electron density is deconvolved from the thermally smeared density, since such a procedure provides estimates of the ADPs minimally biased by the model of the static density. This is what Hirshfeld atom refinement (HAR) is trying to achieve (Jayatilaka & Dittrich, 2008 ▸; Capelli *et al.*, 2014 ▸). A high-level theoretical calculation is first carried out to obtain an accurate static electron density for the unit cell of the crystal. This density is subsequently divided into atomic fragments using the stockholder principle (Hirshfeld, 1977 ▸), which estimates the atomic contributions to the total density at a certain point from the atomic contributions to the procrystal (IAM) density at that point. The resulting Hirshfeld atoms are aspherical and overlapping in space; their straightforward Fourier transforms, the Hirshfeld atom scattering factors, are used to refine the atomic positions and ADPs in standard crystallographic procedures. The two-step procedure of calculating the electron density and then refining the coordinates and ADPs is iterated to convergence. If necessary, the crystal field can be simulated by a cluster of point charges and dipoles surrounding the chemical entity of interest. The aspherical atomic scattering factors applied in HAR enable an accurate localization of hydrogen atoms, eliminate other asphericity shifts and provide ADPs for hydrogen atoms, even if the X-ray data are of medium to low resolution (Capelli *et al.*, 2014 ▸; Woińska *et al.*, 2016 ▸). However, the accuracy of HAR-derived ADPs has not been probed rigorously so far, which highlights the urge for a careful comparison between HAR-, MM- and neutron-derived ADPs.

The downside of HAR is the high computational cost associated with the repeated theoretical calculations of the static electron density. High levels of theory and the inclusion of a self-consistently calculated cluster of charges and dipoles to account for the crystal environment lead to long computation times. Recently, the first HAR based on Hirshfeld atoms calculated from a periodic wavefunction was performed on urea (Wall, 2016 ▸). This should lead to even higher accuracy, but it also implies an even higher computational cost. Capelli *et al.* (2014 ▸) recommended the BLYP/cc-pVTZ level of theory for accurate HAR results at an acceptable cost. So far, it has not been investigated in detail whether a minimal HAR, *i.e.* a single-point calculation on the isolated formula unit, thus *not* using a cluster of charges around the molecule, with low to medium levels of theory is sufficient to give satisfactory hydrogen–element bond distances and ADPs close to neutron diffraction results for all atoms.

A minimal HAR can be performed in a drastically reduced computation time with the new *HARt* (HAR terminal) program introduced in this study – either within a terminal environment or with its implementation in the *Olex2* software (Dolomanov *et al.*, 2009 ▸). If a minimal HAR provides accurate ADPs, then the method opens up a whole new focus in crystallography, where accurate diffraction measurements are used to obtain insight into the thermal behaviour of solids rather than for probing the static electron density, which is represented to the desired level of accuracy by the theoretical calculations. If minimal HARs are found to give element–hydrogen bonds and ADPs nearly as accurate as more elaborate HARs, their combination with the *HARt*–*Olex2* interface is a milestone towards the general applicability of HAR in routine crystallographic studies. The *HARt*–*Olex2* interface is easily accessible due to the widespread use of *Olex2* as mainstream crystallographic software.

The focus of the present paper is to probe the accuracy and precision of the HAR approach and thereby assess its general applicability in crystallographic studies. So far, HAR bond distances and ADPs have been compared with neutron ADPs in some detail only for the dipeptide glycyl-l-alanine at 12, 50, 150 and 295 K (Capelli *et al.*, 2014 ▸). Here, we test the HAR approach on three chemically quite different molecular crystals for which very high-quality single-crystal X-ray and neutron diffraction data are available. The structures investigated in this study are: the aromatic hydro­carbon molecule rubrene (ortho­rhombic 5,6,11,12-tetra­phenyl­tetracene; Jørgensen *et al.*, 2014 ▸; Hathwar *et al.*, 2015 ▸); a co-crystal of a betaine zwitterion, two imidazolium cations and two picrate anions (BIPa) (Overgaard *et al.*, 1999 ▸, 2001 ▸; Jørgensen *et al.*, 2014 ▸); and the salt potassium hydrogen oxalate (KHOx) (Macchi *et al.*, 2000 ▸). HARs for rubrene, BIPa and KHOx have been carried out through the *HARt*–*Olex2* interface with and without the use of cluster charges and dipoles at the HF/def2-SVP and HF/def2-TZVP levels of theory. The hydrogen and non-hydrogen ADPs and the hydrogen–element bond distances obtained from the HARs are compared with those obtained from high-quality neutron data collected at the same temperatures. The accuracy and precision of the HAR results are also evaluated relative to the MM and IAM results. Moreover, it is investigated whether a minimal HAR performs as adequately as more elaborate HARs.

## 2. The *HARt* program   

### 2.1. Implementations   

*HARt* performs Hirshfeld atom refinements, and is available for Linux, Windows and Mac operating systems. It can be downloaded as part of the *Tonto* software package on github (https://github.com/dylan-jayatilaka/tonto), where detailed instructions for the installation procedure are given. There are currently two ways to operate *HARt*, either within a terminal environment or with the pre-installed *HARt* interface implemented in *Olex2*.

For operating *HARt* in the terminal environment, the user must provide an *hkl* file (standard *F*, standard *F* ^2^, *SHELX* *F* or *SHELX* *F* ^2^ format) and a crystallographic information file (CIF) that contains the starting geometry and ADPs of the crystal fragment as obtained in a preceding IAM refinement (*e.g.* with *SHELXL*; Sheldrick, 2015 ▸). The crystal fragment as specified in the CIF is used to calculate the wavefunction in the electron-density step of HAR, so for *Z*′ < 1 and for network compounds (such as salts) the crystal fragment needs to exceed the asymmetric unit. Once CIF and *hkl* files are provided, the user has the following options when starting HAR:

(i) Choice of self-consistent field (SCF) method [either restricted Hartree–Fock (rhf) or Kohn–Sham (rks/BLYP)] and one of the implemented basis sets (STO-3G, def2-SVP, cc-pVDZ, def2-TZVP, cc-pVTZ, def2-TZVPP or cc-pVQZ). The larger of these basis sets are certainly adequate for the vast majority of standard quantum-chemistry property calculations, whereas STO-3G is absolutely un­suitable for producing reliable structural data and should only be used for tests (Table 1 ▸).

(ii) Inclusion of a cluster of charges and dipoles during the SCF calculation to account for the crystal environment. A cluster radius, to be specified *via* the terminal, determines the size of the cluster. Setting the radius to zero disables cluster charges.

(iii) Anisotropic, isotropic or fixed ADPs (refinement of coordinates only) for hydrogen atoms.

(iv) Different criteria for pruning reflections in the refinement process.

(v) Choice of anomalous dispersion correction if the values for *f*′ and *f*′′ are given as further input to *HARt* (Krzeszczakowska *et al.*, 2018 ▸).

The help prompt lists additional details (hart -help). Once all options have been specified, the *hkl* file and CIF are provided, and HAR is initialized, no further user interaction is required. An output file is printed for the user to inspect the progress of HAR and, after completion of HAR, the refined positions and ADPs are printed in a new CIF.

**Figure 1:**
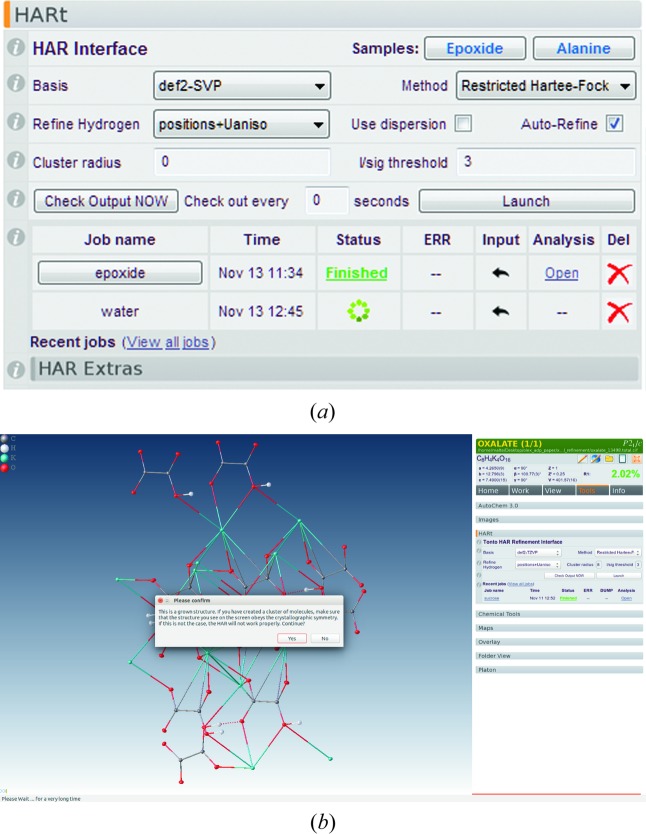
The *HARt*–*Olex2* interface. (*a*) The *HARt* panel in the *Olex2* software, as of 13 November 2017. (*b*) A screenshot of the *Olex2* software, showing the cluster of KHOx used for HAR and a pop-up window asking the user to confirm before starting the refinement.

Compared with the terminal version, the *HARt*–*Olex2* interface (Fig. 1 ▸) comes with some convenient advantages. *Olex2* prepares most of the *HARt* input once a traditional structure refinement has been completed. It offers a graphical user interface (GUI) to input the few options that have to be specified by the user (Fig. 1 ▸ *a*), accessible through the ‘Tools’ panel (Tools > *HARt*). Only the most essential options need to be specified: the quantum-chemical method, the basis set, the cluster radius, the treatment of the hydrogen-atom ADPs, and details about anomalous dispersion correction. A minimal HAR (restricted Hartree–Fock without cluster charges) is the default setting and usually a good starting point. *Olex2* tests whether certain prerequisites required for running a *HARt* job are fulfilled. For example, for structures with *Z*′ < 1 there is a warning to complete the molecule before launching the refinement. This can easily be done using the ‘Grow’ option in *Olex2*, which also allows clusters of any size to be constructed and then used as input into *HARt* (Fig. 1 ▸ *b*).

To run the program from the GUI the ‘Launch’ button is clicked. The *HARt* process starts as an independent named thread. *Olex2* does not monitor the process, but a click on the ‘Check Output Now’ button will check the output directory for any progress and display it in the GUI. Once a *HARt* cycle has been completed, the job name of the process turns into a link and a CIF becomes available for viewing. Using the link, the *HARt* result may be displayed in *Olex2*. All *HARt* jobs are run from (and saved to) a location in the user’s *Olex2* data directory – follow the ‘View all jobs’ link to see them all. To remove jobs from the GUI, simply delete (or archive) the unwanted directory and it will no longer appear in *Olex2*. Depending on the complexity of the structure, a *HARt* refinement can take a very long time, but *Olex2* (and the computer) remain fully usable throughout this time for other tasks. A short video of how to run *HARt* from *Olex2* is available from the *Olex2* YouTube channel at http://bit.ly/2g1tZWj.

### 2.2. Limitations of *HARt*   

Due to the small amount of user interaction it requires and the possibilities that it offers, HAR has the potential to establish itself as a standard crystallographic technique, although at the present state of development some standard procedures are still missing. Extinction corrections are not available currently, but they will be introduced in due course. For anomalous dispersion correction, procedures have been coded, validated and activated inside *HARt* already, and we will report on the implemented procedure in a forthcoming publication (Krzeszczakowska *et al.*, 2018 ▸), which will also cover refinement of anharmonic motions. All refinements are carried out against structure factor magnitudes *F*, not *F* ^2^.

We discourage the use of the current version of *HARt* on systems containing transition metal atoms. For such systems robust *ab initio* wavefunctions are not always available, because they often have low-lying electronic excited states which make convergence of the SCF calculations difficult. We also discourage the use of HAR for structures containing heavy elements. Neglect of relativistic effects may distort the electron density and thus lead to inappropriate aspherical atomic scattering factors. There is also a lack of adequate all-electron basis sets for heavy atoms and the large number of electrons may impede SCF convergence. Effective core potential methods are useless for HAR, because they do not contain explicit core electron densities and can therefore not provide the required atomic form factors. Table 1 ▸ shows a list of basis sets which are available for use with HAR.

## 3. Experimental   

### 3.1. Data   

The X-ray data for rubrene, BIPa and KHOx were taken from previous work that compared MM and neutron ADPs (Jørgensen *et al.*, 2014 ▸; Macchi *et al.*, 2000 ▸). The neutron data sets were measured at the same temperatures as the X-ray data sets for rubrene and BIPa (Jørgensen *et al.*, 2014 ▸), but at a slightly higher temperature (15 *versus* 11 K) for KHOx (Macchi *et al.*, 2000 ▸). Table 2 ▸ lists the crystallographic information and measurement details for the three X-ray and neutron data sets. Pertinent details of the measurements can be found in the original publications.

### 3.2. Challenges for HAR   

The structures considered in this study pose different challenges for HAR:

(i) For rubrene, only a quarter of the molecule is in the asymmetric unit (*Z*′ = 0.25), but initial coordinates and ADPs of the complete molecule are required as an input for the wavefunction calculation. Since HAR uses local non-periodic molecular wavefunctions, the shape of the Hirshfeld atoms is drastically impaired if the theoretical electron density is not calculated from the complete molecule.

(ii) BIPa contains five separate molecules in its asymmetric unit, namely two picrate anions, two imidazolium cations and a betaine zwitterion. HAR is not very well suited to a system comprised of more than one molecule in the asymmetric unit, because the molecular environments for the various independent ions are not modelled in a uniform way – some ions will be surrounded on one side by other ions and on another side by point charges or by neither. Further, if wavefunctions for clusters of molecules are used, it has been observed that SCF convergence and accuracy problems may arise (Woińska *et al.*, 2014 ▸).

(iii) In KHOx, the hydrogen oxalate units are linked *via* strong O—H⋯O hydrogen bonds, but these are neglected if the theoretical electron density is calculated for the isolated formula unit. Consequently, neither a minimal HAR nor perhaps a HAR with cluster charges can be expected to give an accurate O—H bond distance and accurate hydrogen ADPs. Building a cluster of neighbouring molecules around the formula unit introduces a hydrogen bond into the quantum-chemical calculation and this approach might thus be expected to yield more accurate hydrogen parameters. However, taking the long-range electrostatic interactions between the ions into account might require a periodic treatment, as performed by Wall (2016 ▸).

### 3.3. Refinements   

**Figure 2:**
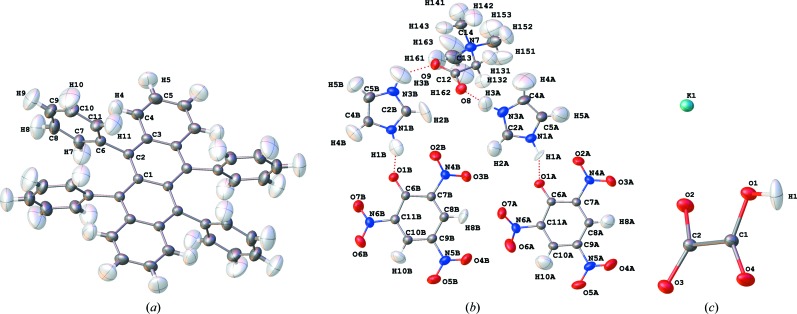
Molecular structures and anisotropic displacement parameters (90% probability surfaces) for (*a*) rubrene, (*b*) BIPa and (*c*) KHOx, obtained from HAR and plotted with *Olex2* (HF/def2-TZVP, with point charges and dipoles simulating the crystalline environment for rubrene and BIPa, or an explicit cluster of neighbouring molecules for KHOx). Corresponding representations based on the neutron data are shown in Fig. S3 in the supporting information.

HARs were performed with the *HARt* interface in *Olex2* using the restricted Hartree–Fock method (rhf or HF) with two different basis sets for each structure: HF/def2-SVP (adequate level of theory, Table 1 ▸) and HF/def2-TZVP (excellent level of theory, Table 1 ▸). The geometry of a *SHELXL* IAM refinement served as input. For rubrene, a complete molecule comprising four asymmetric units was used in the wavefunction calculations (Fig. 2 ▸ *a*). For BIPa, the asymmetric unit consisting of the five co-crystallized ions was considered as a supermolecule and used for the wavefunction calculation. To minimize the bias on the ADPs, the ions in the supermolecule were chosen so that the strongest intermolecular interactions (hydrogen bonds N1*A*/*B*—H1*A*/*B*⋯O1*A*/*B* and N3*A*/*B*—H3*A*/*B*⋯O8/9, Fig. 2 ▸ *b*) are within the asymmetric unit and hence within the wavefunction. The HF/def2-TZVP calculations on rubrene and BIPa were performed both with and without a cluster of charges and dipoles, simulating the crystal field of all neighbouring mol­ecules that have any atom within an intermolecular distance of 8 Å (from now on referred to as ‘charges’). For KHOx, HAR with the def2-TZVP basis set was performed with and without an explicit cluster of hydrogen oxalate and potassium ions built around the formula unit, obeying the crystallographic symmetry (Fig. 1 ▸ *b*; from now on referred to as a ‘cluster’). This cluster was obtained with the ‘Grow’ option in *Olex2* and used in *HARt* for the wavefunction calculation. From the static electron density of the cluster, aspherical atomic scattering factors were obtained for the asymmetric unit atoms, the only ones included in the structure refinement step of HAR. The hydrogen atoms were refined freely and anisotropically in all HARs (see Figs. 1 ▸ *a* and 2 ▸). For the refinements without an implicit or explicit cluster at a low basis set, HARs took between several minutes to hours on our standard laboratory desktop computers, whereas they took several days with higher basis sets and explicit or implicit clusters.

To enable conclusive comparisons between HAR and multipolar refinements, the latter were redone using *XD2006* (Volkov *et al.*, 2006 ▸) using the exact same reflections as used in HAR, together with the local site symmetries, constraints and κ-treatments given in the original publications (Jørgensen *et al.*, 2014 ▸; Macchi *et al.*, 2000 ▸). In these refinements the hydrogen–element bond lengths were fixed to the distances obtained from the neutron diffraction experiments and the hydrogen ADPs estimated with the SHADE approach (Madsen, 2006 ▸). Additionally, for rubrene the hydrogen ADPs were constrained to the values from the neutron diffraction experiment to test whether this constraint would change the non-hydrogen ADPs compared with those obtained with hydrogen ADPs estimated using SHADE. Details of this comparison are deposited with the supporting information. For all three compounds, a further multipole model (MM) was refined without any constraints from the neutron diffraction experiments, *i.e.* hydrogen-atom positions and their isotropic displacement parameters were refined freely. From these refinements only the hydrogen–element bond lengths are discussed. All other comparisons discussed below refer to MMs with fixed hydrogen bond lengths and SHADE ADPs.

## 4. Results and discussion   

### 4.1. Comparison of *R* factors and residual density representations   

*R* factors, which measure the agreement between calculated and observed structure factors, provide an initial overall indication of the accuracy of the models (Table 3 ▸). For all structures, the IAMs have the highest *R*_1_ and *wR*_2_ factors. Both the MMs and HARs give substantially lower values for rubrene and BIPa and slightly lower values for KHOx. In the IAM the highest residual electron densities are associated with bonds, lone pairs and other aspherical features (Table 3 ▸). Aspherical atoms account for these features and decrease the *R* factors correspondingly.

**Figure 3:**
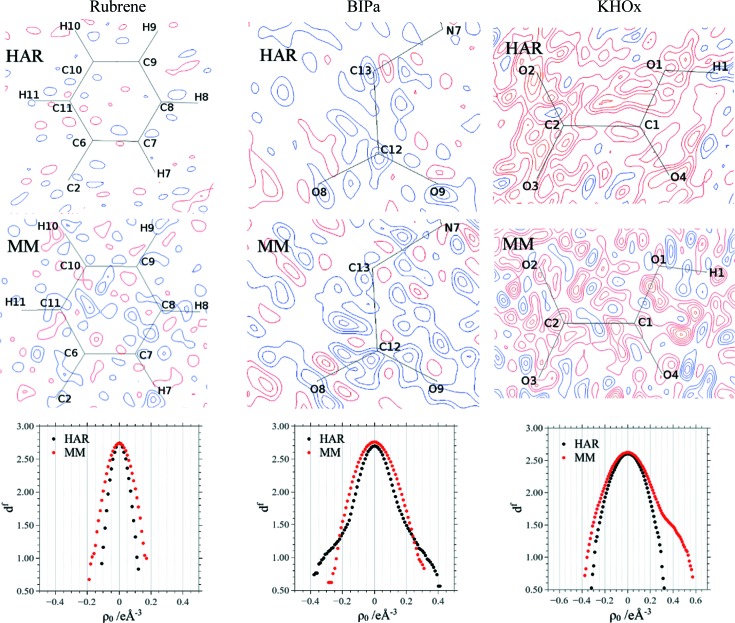
Residual density plots for rubrene, BIPa and KHOx of the HARs (top row) and the MMs (middle row) calculated with the full resolution and the observed reflections as given in Table 2 ▸. Blue denotes positive and red negative, and the contour interval is 0.05 e Å^−3^. The bottom row shows Henn–Meindl fractal dimension plots based on the complete unit-cell electron density. Additional cut planes through other ions of BIPa are given in the supporting information. They show the same trends.

The *R*_1_ factors of the MMs are always slightly lower and the *wR*_2_ factors are slightly or significantly (BIPa) higher than those of the HAR models. Overall, the residual density distributions resulting from HAR show fewer features than the MM maps (Fig. 3 ▸, and Fig. S1 in the supporting information). Minimum and maximum residual density values are slightly lower in the HAR models of rubrene and KHOx but higher for BIPa. Neither model shows systematic accumulation of positive residual density on the bonds.

Henn–Meindl plots correlate the residual density with its fractal dimension; they show parabolas centred at zero if a model accounts for the data (Meindl & Henn, 2008 ▸). The plots for HAR and MM of rubrene are nearly parabolic, with the former being slightly sharper, in accordance with the slightly lower maximum and minimum residual density peaks of the HAR model (Table 3 ▸). For BIPa, MM yields a sharper and more parabolic plot than HAR, although the curve for HAR is still symmetric (Fig. 3 ▸). The deviation from the parabolic shape in HAR can be attributed to the fact that HAR is not ideally suited for *Z*′ > 1 structures nor for treating disorder, which is present in the betaine molecule to a minor extent (compare discussion relating to Fig. 6 below). For KHOx, the Henn–Meindl plot indicates an unmodelled positive electron density in MM (shoulder in the red curve), which is however successfully modelled in HAR, leading to a near-ideal parabolic curve centred around zero.

We recall that the electron density obtained from MM represents a fit to the experimental structure factors, whereas the electron density used in HAR originates from a quantum-mechanical calculation. The fact that the HAR treatments lead to lower and more even residual density distributions indicates that, for small organic molecules, the theoretical static electron density is more suitable for reconstructing the experimental diffraction pattern accurately than an experimental electron-density model.

In spite of the encouraging results presented so far, there are a number of shortcomings of the present implementation of HAR. There is information in the experimental X-ray diffraction data that is not modelled in the theoretical static electron density at the Hartree–Fock level, *e.g.* electron correlation (Genoni *et al.*, 2017 ▸) or polarization (Grabowsky *et al.*, 2017 ▸), and there are relativistic effects if heavier elements are involved (Bučinský *et al.*, 2016 ▸). There are several ways of mitigating these weaknesses. One is to switch to higher-level *ab initio* models which account for some of the shortcomings. Another way is X-ray constrained wavefunction fitting (XCW), which refines the orbital coefficients against the experimental structure factors with the atomic positions and ADPs fixed at the HAR level (Jayatilaka & Grimwood, 2001 ▸). The combination of HAR and XCW is called X-ray wavefunction refinement (Grabowsky *et al.*, 2012 ▸). This approach generally yields lower *R* factors than an MM and, simultaneously, a better agreement with the electron-density topology from benchmarking theoretical calculations (Woińska *et al.*, 2017 ▸). A sufficiently well parameterized MM will also extract this information from the X-ray data, since it does not depend on assumptions inherent in any level of electronic structure theory.

### 4.2. Comparison of anisotropic displacement parameters   

Fig. 2 ▸ shows the anisotropic displacement parameters with 90% probability surfaces for rubrene, BIPa and KHOx as obtained from HAR (rhf/def2-TZVP with charges or cluster). The 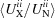 and 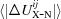 values for the hydrogen and non-hydrogen ADPs of rubrene, BIPa and KHOx are listed in Table 4 ▸. 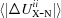 is reported alongside 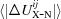 because the off-diagonal ADPs are generally small in magnitude and their differences tend to conceal deviations of the diagonal ADPs from the corresponding neutron values. Assuming that the neutron data can be considered as a true reference, these numbers are a measure of the accuracy of the different X-ray refinement models, while the corresponding sample standard deviations after averaging are a measure of their precision. Since the neutron data are also affected by experimental errors, it is also sensible to report the root-mean-squared differences of X-ray and neutron ADPs weighted by the combined standard uncertainties (csu) (Schwarzenbach *et al.*, 1995 ▸)
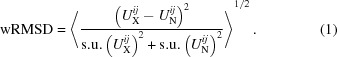
As may be seen from the tables in Section 3 of the supporting information and the respective CIFs, the standard uncertainties of the X-ray ADPs for non-hydrogen atoms tend to be comparable with, and slightly lower than, the neutron ADPs, whereas those for the hydrogen atoms are one order of magnitude larger than those of the neutron ADPs. Thus the X-ray ADP s.u.s dominate the value of the combined standard uncertainty for the hydrogen atoms and the quantity wRMSD is a measure of the accuracy of the X-ray ADPs. wRMSDs were previously employed by Capelli *et al.* (2014 ▸) for the comparison of HAR and neutron ADPs.

X-ray and neutron ADPs are in statistical agreement if wRMSD = 1. Although wRMSDs are generally smaller for MM and HAR models than for IAM, most values are between 1 and 2 (Table 4 ▸). Note, however, that s.u.s are normally underestimated (Kaminski *et al.*, 2014 ▸); for multiple determinations of the same crystal structure, values ranging from 1.5 to 2.0 are not uncommon (Taylor & Kennard, 1983*a* ▸,*b* ▸; Martín & Orpen, 1996 ▸).

For all three compounds, the non-hydrogen IAM ADPs are of lower accuracy and precision than the HAR and MM ADPs (Table 4 ▸). However, all parameters listed for the IAM ADPs in Table 4 ▸ still imply excellent agreement with the neutron data. This finding is largely due to the high resolution and exceptionally high quality of the data and should not be generalized to IAM refinements of data with lower resolution and lower quality. The measures of agreement for the MM and HAR non-hydrogen ADPs are practically the same, indicating comparable accuracy and precision. The high discrepancy between neutron and HAR ADP values reported by Jørgensen *et al.* (2014 ▸) turns out to be due to a HAR input error and a numerical mistake in the averaging procedure. Table 4 ▸ also shows that the results of all HARs are essentially the same with and without cluster charges or an explicit cluster, and the basis set def2-SVP performs as well as the more flexible def2-TZVP basis set.

**Figure 4:**
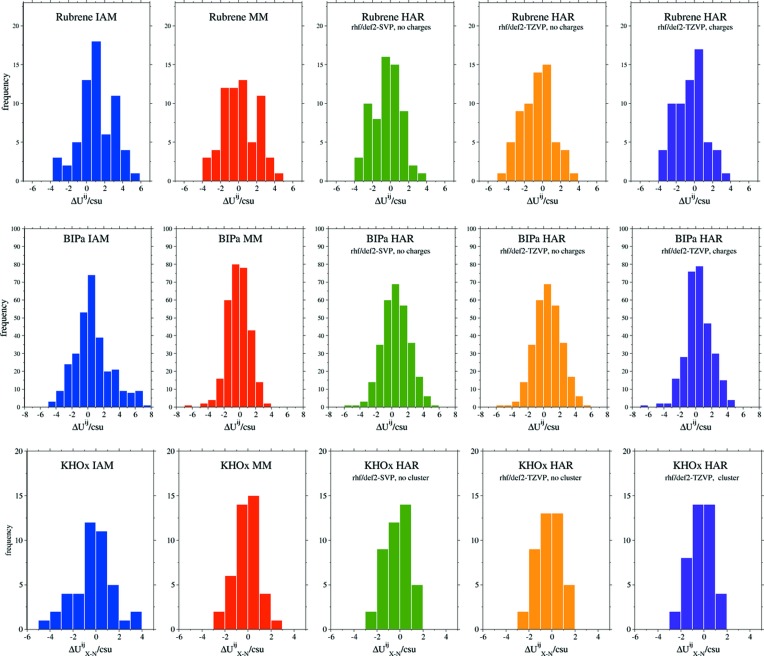
Histograms showing binned ratios 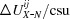 for the non-hydrogen ADPs of rubrene, BIPa and KHOx.

Fig. 4 ▸ shows histograms of binned 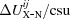 values for rubrene, BIPa and KHOx. They visually demonstrate that non-hydrogen ADPs obtained from MM and HARs are more accurate and precise than those from IAM: the clusters of HAR and MM ADP differences are narrower than those of the IAM ADP differences. No preference of HAR over MM or MM over HAR is evident. The plots suggest no obvious difference between the minimal and more sophisticated HARs.

In summary, two important conclusions can be drawn from the results for the non-hydrogen ADPs in Table 4 ▸ and Fig. 4 ▸: (i) the HAR ADPs are as accurate and precise as the MM ADPs, and (ii) a minimal HAR gives practically the same results as the more elaborate and computationally more expensive HARs. Also, the ADPs for BIPa, a system with five ions in the asymmetric unit, and for KHOx, a network compound, are accurately and precisely determined by all the HARs performed. Thus, the challenges for HAR mentioned in Section 3.2 have been met.

Zhurov *et al.* (2011 ▸) showed that, with exceptionally good data, it is possible to refine hydrogen ADPs within an MM refinement. With HAR, hydrogen atoms can routinely be refined anisotropically, albeit with a substantially lower accuracy and precision than found for the non-hydrogen atoms (Table 4 ▸), and in agreement with previous reports (Capelli *et al.*, 2014 ▸; Woińska *et al.*, 2016 ▸). Alternatively, anisotropic hydrogen ADPs may be estimated with the SHADE procedure, which combines a rigid-body contribution derived from the non-hydrogen atoms (TLS approximation) with a contribution due to *X*–H stretching and bending vibrations taken from a database based on neutron data (Madsen, 2006 ▸). Hydrogen ADPs from the SHADE procedure give lower hydrogen 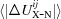 values than those from HAR for rubrene, BIPa and KHOx. This implies that SHADE ADPs are very well suited to multipole modelling, and neither residual densities nor non-hydrogen ADPs are visibly affected by the choice of the hydrogen ADPs (SHADE-estimated or neutron-derived; see discussion in Section 2 of the supporting information). For rubrene, the agreement measures of the hydrogen atoms reveal only minor differences between the different types of HARs performed, while for BIPa, the basis set def2-TZVP gives slightly superior results than the less sophisticated basis set def2-SVP (Table 4 ▸).

The single hydrogen atom in KHOx is linked to a neighbouring hydrogen oxalate unit *via* a strong intermolecular O—H⋯O hydrogen bond in a charged structure. All hydrogen parameters in Table 4 ▸ clearly show that for HAR it is necessary to build an explicit cluster of neighbouring KHOx units around the central formula unit in order to obtain the hydrogen ADPs from the X-ray data with an acceptable accuracy and precision. This is remarkable, because it shows that the experimental X-ray data are sufficient to capture fine details in the electron density, here the polarization of the hydrogen-atom electron density due to the hydrogen-bond interactions. However, a rather high level of theory and a cluster of whole molecules had to be included in the refinement. Calculating the theoretical electron density of just the formula unit results in unacceptably high 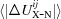 values for the hydrogen ADPs, but not for the non-hydrogen ADPs which seem unaffected by the intermolecular interaction.

**Figure 5:**
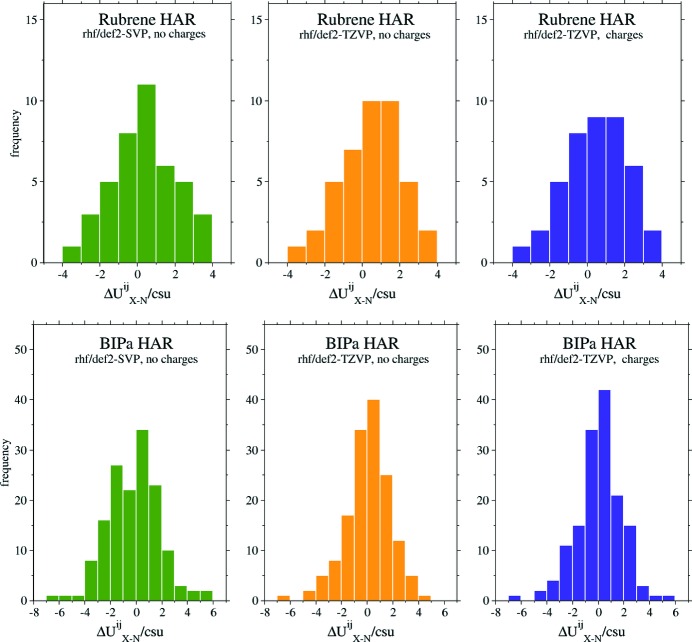
Histograms showing binned ratios of 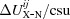 for the hydrogen ADPs of rubrene and BIPa.

Fig. 5 ▸ shows histograms of binned 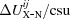 values for the hydrogen ADPs obtained from the HARs of rubrene and BIPa. No data for KHOx are presented because the system has only one hydrogen atom. The histograms indicate that the basis set has only a minor influence on the accuracy and precision of rubrene’s hydrogen ADPs, while for BIPa the influence is more distinct – the more complex def2-TZVP gives more accurate and precise hydrogen ADPs.

**Figure 6:**
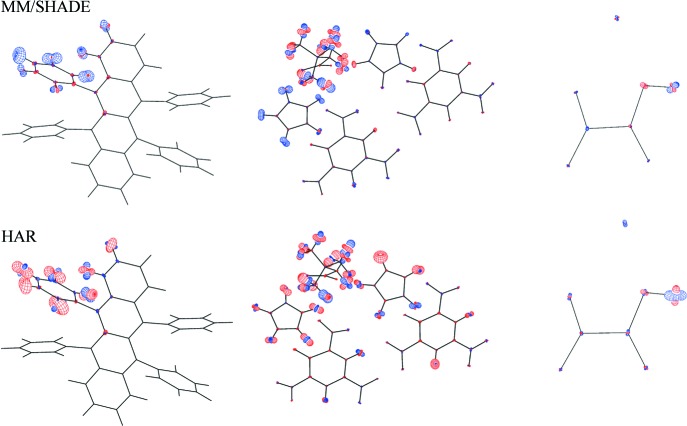
Difference between neutron ADPs and those from MM/SHADE (top) or HAR refinements (bottom) for rubrene (left), BIPa (middle) and KHOx (right). The basis set was rhf/def2-TZVP, with charges or with a cluster, and the plots were drawn using the *PEANUT* software (Hummel *et al.*, 1990 ▸). The plots refer to a 50% probability level of the ADP RMSDs, scaled by a factor of 2. Blue denotes positive and red negative.

Fig. 6 ▸ shows ADP difference plots comparing the MM/SHADE and HAR ADPs (rhf/def2-TZVP, with charges or a cluster) with the neutron ADPs. The plots show that the hydrogen ADPs are determined less accurately than the non-hydrogen ADPs, whose difference ADPs are barely visible. The differences for the hydrogen atoms of rubrene and BIPa based on MM/SHADE values show a tendency to systematic positive differences perpendicular to the *X*—H bonds in the aromatic plane and along the methyl C—H bonds. Therefore, these patterns indicate a systematic shortcoming of the SHADE procedure. By comparison, the differences based on HAR ADP values appear more or less random, indicating that HAR has extracted the (limited) information available in the data. Some minor amount of increased atomic displacement is visible in the methyl groups of the betaine zwitterion in hydrogen and non-hydrogen atoms, which may be due to some dynamic disorder. This is also reflected in the size of the hydrogen ADPs in Fig. 2 ▸(*b*) and the residual density distribution for the betaine group (Fig. 3 ▸). Overall, one may conclude that, if neutron data are unavailable, the best treatment of hydrogen parameters is obtained with the SHADE model, but HAR performs well if one considers that it is solely based on the X-ray diffraction data.

Table S25 in the supporting information shows averages of the differences in mean-squared displacement amplitudes (DMSDAs) along different kinds of bonds (Hirshfeld rigid-bond test; Hirshfeld, 1976 ▸). These numbers provide information on the orientations of the ADPs relative to the bond axes. For all methods (neutron, IAM, MM and different HARs), the DMSDA values of bonds involving only non-H atoms are below the limit of 0.001 Å^2^ suggested by Hirshfeld (1976 ▸). They confirm the excellent quality of both the X-ray and neutron data. For all bond types between non-H atoms, the differences between the averages obtained for the different refinement methods are insignificant. Concerning *X*—H bonds, the neutron values are between 0.0053 and 0.0063 Å^2^, close to the default value of 0.005 Å^2^ (Madsen, 2006 ▸), whereas the SHADE results vary from 0.005 to 0.015 Å^2^ and the HAR results are around 0.015 to 0.020 Å^2^, systematically too big by a factor of 2–3, but with a large dispersion as reflected in the sample standard deviations.

### 4.3. Comparison of hydrogen–element bond distances   

In Table 5 ▸, the hydrogen–element bond distances of rubrene, BIPa and KHOx from the X-ray refinement techniques, *r*_X_, are compared with the corresponding neutron values, *r*_N_. The ratio 〈*r*_X_/*r*_N_〉, the mean absolute differences of *r*_X_ and *r*_N_, 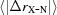, and the corresponding wRMSD measure the average deviation of the X-ray hydrogen–element bond distances from the values determined from neutron data. The values for the IAM clearly indicate the well known underestimation of the hydrogen–element bond distances by 0.11 to 0.19 Å in all structures. This study once again shows that hydrogen–element bond distances from HAR are accurate, as seen from the low 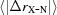 values as well as *r*_X_/*r*_N_ ratios and wRMSD ratios close to unity. The average absolute deviation is about 0.007 Å for rubrene and 0.015 Å for BIPa. For these two structures, the application of cluster charges and the choice of the basis set do not influence the results – a minimal HAR can determine element–hydrogen bond distances with an accuracy equal to more elaborate HARs.

The hydrogen atom in KHOx is involved in a strong intermolecular hydrogen bond, which is disregarded if the electron density is obtained from the isolated formula unit. The O—H bond distances are seen to be underestimated if the HAR is performed without introducing the intermolecular hydrogen bond into the wavefunction used to calculate the aspherical atomic form factors. Building a cluster of potassium and hydrogen oxalate ions around the formula unit gives the most accurate O—H distance of all the HARs performed for KHOx.

In the MM, the hydrogen–element distances are usually constrained either to experimental neutron values or, if the corresponding neutron data are not available, to averaged neutron data. In Table 5 ▸, the parameters from a multipole refinement with unconstrained isotropic hydrogen atoms are listed. The C—H bond distances for rubrene can be determined almost as accurately as with the HARs, albeit with a substantially lower precision. The MM hydrogen–element bond distances of BIPa and KHOx are superior to those obtained from the IAM, but still too short and less precise than the hydrogen–element bond distances of the HARs.

## 5. Conclusions   

It has been shown that the anisotropic displacement parameters from Hirshfeld atom refinement (HAR ADPs) for non-hydrogen atoms in three organic molecular crystals, rubrene, BIPa and KHOx, are as accurate and precise as the ADPs from multipolar refinements or from neutron diffraction data. Both MM and HAR employ aspherical atomic scattering factors and consequently give more accurate ADPs than the IAM. The non-hydrogen and hydrogen 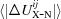 values and 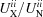 ratios of the HARs are nearly the same in the presence and absence of cluster charges. If HAR is performed only for the asymmetric unit of the network compound KHOx without an explicit cluster around it, reasonable results are obtained for the non-hydrogen elements. However, accurate hydrogen parameters require HAR on the formula unit surrounded by an explicit cluster of neighbouring molecules, emulating the influence of the strong intermolecular O—H⋯O hydrogen bond and charge interactions. In this case the hydrogen HAR ADPs are as accurate as those for molecules without hydrogen-bond interactions between different asymmetric units, and the O—H bond length is only a little less accurate than that from neutron diffraction data. In summary, the compounds chosen in this study posed three challenges for HAR (*Z*′ < 1, *Z*′ > 1 and a periodic network), all of which could be resolved by the strategies presented in this study.

The *R*_1_ and *wR*_2_ factors of the aspherical refinement models are significantly lower than for the IAM, because aspherical features of the electron density are considered. This also shows in lower and more randomly distributed maximum residual density peaks. Furthermore, for the small organic compounds considered here, the static electron density used in HAR as calculated from the quantum-mechanical *ansatz* is accurate enough to reconstruct the measured structure factors as successfully as a multipole model and derive non-hydrogen ADPs with the same accuracy and precision as from a multipole model, whose density results from a fit to the experimental structure factors. Moreover, HAR has the advantage of allowing accurate modelling of hydrogen ADPs and hydrogen–element bond distances, which MM does not.

Hirshfeld atoms by construction have a free spherical atom bias, so they will tend to be less charged and less polarized than might be expected from formal charges or from other partitioning schemes (Bultinck *et al.*, 2009 ▸). This may affect the derived ADPs. Likewise, it is assumed that the partitioned atomic density does not change when atoms undergo thermal motion. The influence of these effects on the accuracy of HAR-derived ADPs has not been probed so far, but is expected to be small.

For many systems a basis set of moderate quality, such as def2-SVP, gives ADPs and hydrogen–element distances as accurate as those from higher basis sets but at a lower computational cost. In fact, bond distances involving hydrogen atoms obtained with moderate quality basis sets are as accurate as those from neutron diffraction data, provided no exceptionally strong intermolecular interactions are present. Such minimal HARs (*e.g.* HF/def2-SVP without cluster charges) can be performed on average machines overnight, even for larger systems such as BIPa and rubrene. With the *HARt*–*Olex2* interface, HAR can be performed with little effort following a conventional structure refinement in the *Olex2* software.

## 6. Supporting information   

The supporting information document includes four sections: (1) Residual density representations in different molecular planes, (2) Discussion of a multipole model with hydrogen ADPs taken from the neutron diffraction results, (3) Individual *U^ij^* values for all atoms in all models, and (4) Hirshfeld rigid-bond tests.

CIFs of the highest-quality HAR models (HF/def2-TZVP level with charges/cluster) are deposited with the Cambridge Structural Database under the refcodes 1565217 to 1565219. They can be obtained free of charge *via* https://www.ccdc.cam.ac.uk/structures/.

In addition, CIFs of all IAMs, HARs and MMs are deposited with the supporting information for this paper.

## Supplementary Material

Crystal structure: contains datablock(s) I. DOI: 10.1107/S2052252517015548/lc5093sup1.cif

CIF information for BIPa in a zipped archive. DOI: 10.1107/S2052252517015548/lc5093sup2.zip

CIF information for rubrene in a zipped archive. DOI: 10.1107/S2052252517015548/lc5093sup3.zip

CIF information for KHOx in a zipped archive. DOI: 10.1107/S2052252517015548/lc5093sup4.zip

Additional figures and tables. DOI: 10.1107/S2052252517015548/lc5093sup5.pdf

Supporting information file. DOI: 10.1107/S2052252517015548/lc5093sup6.cml

CCDC references: 1565217, 1565218, 1565219

## Figures and Tables

**Table 1:** Basis sets available in the program *HARt*

Testing	Adequate	Excellent	Benchmark	Availability
STO-3G	def2-SVP	def2-TZVP	def2-TZVPP	H-Kr
	cc-pVDZ	cc-pVTZ	cc-pVQZ	H-Kr (no K)

**Table 2:** Crystallographic information and measurement details of rubrene, BIPa and KHOx The first column for each compound refers to X-ray data and the second column to neutron measurements.

	Rubrene (Jørgensen *et al.*, 2014 ▸)		BIPa (Jørgensen *et al.*, 2014 ▸)		KHOx (Macchi *et al.*, 2000 ▸)	
Empirical formula	C_42_H_28_		C_25_N_11_O_16_H_25_		KHC_2_O_4_	
Crystal system	Orthorhombic		Monoclinic		Monoclinic	
Space group	*Cmce*		*C*2/*c*		*P*2_1_/*c*	
λ (Å)	0.7107	0.4–3.4†	0.7107	0.4–3.4†	0.5616	1.008
*a* (Å)	26.8106 (3)	26.7972 (3)	33.5939 (5)	33.5759 (1)	4.265 (1)	4.267 (1)
*b* (Å)	7.1602 (1)	7.1617 (1)	7.6658 (1)	7.6607 (1)	12.796 (1)	12.816 (7)
*c* (Å)	14.2029 (1)	14.1940 (2)	25.1324 (3)	25.1114 (2)	7.490 (1)	7.501 (6)
α (°)	90	90	90	90	90	90
β (°)	90	90	114.716 (2)	114.6982 (4)	100.77 (1)	100.82 (6)
γ (°)	90	90	90	90	90	90
*T* (K)	100 (1)	100 (1)	100 (1)	100 (1)	11 (1)	15 (1)
sin(θ)/λ_max_ (Å^−1^)	1.1	1.25	1.1	1.0	1.4	0.8
*R*_int_	0.0328	N/A†	0.0381	N/A†	0.0169	0.0585
*N*_meas_, *N*_uniq_	83536, 7703	98478, N/A	41957, 31489	73225, N/A	12997, 4911	2991, 1436
*N*_obs_ (*F* > 3σ)‡	6457	22775	23751	25886	4439	1082

† Data from Laue time-of-flight neutron diffraction.

‡ *F* > 4σ for *SHELXL* refinements, so the numbers of observed reflections differ; see CIFs deposited as supporting information.

**Table 3:** Refinement results of the IAMs, MMs and HARs of rubrene, BIPa and KHOx. All values refer to the full resolutions of the data sets and the observed reflections as specified in Table 2 ▸

Compound	Method	*R*_1_	*wR*_2_	Δρ_min/max_ (e Å^−3^)
Rubrene	IAM	0.0418	0.1305	−0.23/0.67
	MM	0.0245	0.0565	−0.19/0.18
	HAR rhf/def2-svp, no charges	0.0262	0.0405	−0.11/0.13
	HAR rhf/def2-tzvp, no charges	0.0259	0.0400	−0.11/0.13
	HAR rhf/def2-tzvp, charges	0.0256	0.0395	−0.11/0.13
				
BIPa	IAM	0.0523	0.1281	−0.33/0.75
	MM	0.0347	0.0742	−0.29/0.32
	HAR rhf/def2-svp, no charges	0.0368	0.0324	−0.40/0.42
	HAR rhf/def2-tzvp, no charges	0.0366	0.0322	−0.39/0.43
	HAR rhf/def2-tzvp, charges	0.0365	0.0321	−0.40/0.41
				
KHOx	IAM	0.0221	0.0603	−0.77/0.72
	MM	0.0181	0.0402	−0.39/0.58
	HAR rhf/def2-svp, no cluster	0.0198	0.0332	−0.33/0.35
	HAR rhf/def2-tzvp, no cluster	0.0195	0.0329	−0.32/0.32
	HAR rhf/def2-tzvp, cluster	0.0196	0.0321	−0.32/0.32

**Table 4:** Comparison of X-ray and neutron ADPs for rubrene, BIPa and KHOx from IAM, MM and HAR models 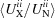
 is the mean ratio of the diagonal X-ray and neutron ADPs. 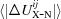 and 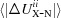 are the mean absolute differences between X-ray and neutron ADPs (units Å^2^). wRMSD is the weighted root-mean-squared difference as defined in equation (1). Charges = a cluster of point charges and dipoles. Cluster = an explicit cluster of ions around the central ion pair. Values in brackets are the sample standard deviations.

		Non-hydrogen				Hydrogen			
Compound	Method	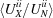	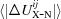	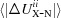	wRMSD†	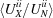	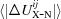	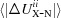	wRMSD†
Rubrene	IAM	1.02 (1)	0.00027 (19)	0.00031 (18)	2.27				
	MM (non-H ADPs)/SHADE (H ADPs)	1.01 (3)	0.00021 (17)	0.00026 (19)	1.84	0.98 (6)	0.0023 (19)	0.0027 (24)	‡
	HAR rhf/def2-svp, no charges	1.00 (2)	0.00019 (15)	0.00023 (17)	1.65	1.07 (19)	0.0045 (32)	0.0054 (33)	1.73
	HAR rhf/def2-tzvp, no charges	0.99 (2)	0.00020 (17)	0.00024 (17)	1.72	1.12 (19)	0.0046 (32)	0.0056 (33)	1.69
	HAR rhf/def2-tzvp, charges	0.99 (2)	0.00020 (16)	0.00024 (18)	1.68	1.11 (19)	0.0045 (32)	0.0050 (31)	1.69
									
BIPa	IAM	1.05 (6)	0.00062 (52)	0.00081 (60)	2.38				
	MM (non-H ADPs)/SHADE (H ADPs)	0.99 (3)	0.00037 (31)	0.00042 (34)	1.10	1.02 (23)	0.0045 (51)	0.0052 (49)	‡
	HAR rhf/def2-svp, no charges	1.03 (4)	0.00042 (33)	0.00052 (37)	1.80	1.06 (41)	0.0089 (76)	0.0098 (82)	1.92
	HAR rhf/def2-tzvp, no charges	1.02 (4)	0.00039 (30)	0.00047 (34)	1.68	1.13 (35)	0.0078 (60)	0.0088 (65)	1.68
	HAR rhf/def2-tzvp, charges	1.02 (4)	0.00040 (30)	0.00048 (30)	1.69	1.11 (35)	0.0078 (59)	0.0090 (62)	1.74
									
KHOx	IAM	0.98 (8)	0.00048 (44)	0.00035 (37)	1.80				
	MM (non-H ADPs)/SHADE (H ADPs)	0.99 (9)	0.00030 (27)	0.00040 (33)	1.03	0.93 (1)	0.0018 (10)	0.0011 (25)	‡
	HAR rhf/def2-svp, no cluster	0.99 (10)	0.00032 (31)	0.00041 (36)	1.14	3.57 (389)	0.0298 (414)	0.0457 (582)	3.64
	HAR rhf/def2-tzvp, no cluster	0.99 (10)	0.00033 (31)	0.00042 (37)	1.16	3.07 (307)	0.0238 (332)	0.0354 (474)	3.35
	HAR rhf/def2-tzvp, cluster	0.97 (10)	0.00032 (29)	0.00041 (36)	1.03	0.83 (65)	0.0059 (39)	0.0089 (30)	1.21

† The standard uncertainties are used for calculating the wRMSD values.

‡ Since SHADE ADPs are estimated from tabulated and calculated data, they contain no standard uncertainties, so no wRMSD can be calculated.

**Table 5:** Comparison of the element–hydrogen bond lengths *r*(*X*—H) obtained from the X-ray refinement models (IAM, MM and HAR) with the values obtained from neutron refinements The average bond lengths are given by 〈*r*(*X*—H)〉 (Å). 〈*r*_X_/*r*_N_〉 is the average ratio of the X-ray and neutron bond lengths, 〈|Δ*r*_X—N_|〉 (Å) is their mean average difference, and wRMSD is the weighted root-mean-squared deviation [equation (1)]. Values in brackets are the sample standard deviations.

Compound	Method	Bond type	〈*r*(*X*—H)〉	〈*r*_X_/*r*_N_〉	〈|Δ*r*_X—N_|〉	wRMSD†
Rubrene	Neutron (aniso)	C—H	1.086 (1)			
	IAM (iso)	C—H	0.981 (28)	0.90 (3)	0.105 (28)	9.51
	MM (iso)	C—H	1.101 (35)	1.01 (3)	0.032 (19)	1.24
	rhf/def2-svp (aniso)	C—H	1.082 (8)	1.00 (1)	0.007 (5)	1.32
	rhf/def2-tzvp, no charges (aniso)	C—H	1.082 (7)	1.00 (1)	0.007 (4)	1.35
	rhf/def2-tzvp, charges (aniso)	C—H	1.084 (8)	1.00 (1)	0.007 (3)	1.18
						
BIPa	Neutron (aniso)	C—H	1.084 (5)			
	IAM (iso)	C—H	0.935 (40)	0.86 (4)	0.149 (39)	14.18
	MM (iso)	C—H	1.021 (78)	0.94 (7)	0.073 (70)	3.77
	rhf/def2-svp, no charges (aniso)	C—H	1.083 (23)	1.00 (2)	0.016 (13)	2.02
	rhf/def2-tzvp, no charges (aniso)	C—H	1.076 (21)	0.99 (2)	0.016 (13)	2.08
	rhf/def2-tzvp, charges (aniso)	C—H	1.077 (22)	0.99 (2)	0.016 (13)	1.98
						
	Neutron (aniso)	N—H	1.045 (16)			
	IAM (iso)	N—H	0.861 (74)	0.82 (6)	0.184 (64)	16.16
	MM (iso)	N—H	0.948 (93)	0.91 (9)	0.098 (101)	2.04
	rhf/def2-svp, no charges (aniso)	N—H	1.058 (30)	1.01 (1)	0.014 (11)	1.70
	rhf/def2-tzvp, no charges (aniso)	N—H	1.053 (22)	1.01 (1)	0.012 (5)	1.24
	rhf/def2-tzvp, charges (aniso)	N—H	1.050 (22)	1.00 (1)	0.009 (5)	1.03
						
KHOx	Neutron (aniso)	O—H	1.060			
	IAM (iso)	O—H	0.866‡	0.82‡	0.192‡	‡
	MM (iso)	O—H	0.914	0.86	0.146	
	rhf/def2-svp, no cluster (aniso)	O—H	1.009	0.95	0.051	
	rhf/def2-tzvp, no cluster (aniso)	O—H	1.012	0.96	0.048	
	rhf/def2-tzvp, cluster (aniso)	O—H	1.044	0.98	0.016	

† The standard uncertainties are used for calculating the wRMSD values.

‡ Since there is only one *X*—H bond in KHOx, no sample standard deviation and no wRMSD values can be calculated for any X-ray model.
