# Supplementary material for: Probing the accuracy and precision of Hirshfeld atom refinement with HARt interfaced with Olex2
Source: IUCrJ. 2018 Jan 1;5(Pt 1):32–44. doi: 10.1107/S2052252517015548 (PMC5755575; doi:10.1107/S2052252517015548)

checkCIF/PLATON report

You have not supplied any structure factors. As a result the full set of tests cannot be run.

THIS REPORT IS FOR GUIDANCE ONLY. IF USED AS PART OF A REVIEW PROCEDURE FOR PUBLICATION, IT SHOULD NOT REPLACE THE EXPERTISE OF AN EXPERIENCED CRYSTALLOGRAPHIC REFEREE.

No syntax errors found. CIF dictionary Interpreting this report

Datablock: bipa_tzvp_c

Bond precision: C-C = 0.0006 A Wavelength=0.71070

Cell:	a=33.5939(5)	b=7.66580(8)	c=25.1324(3)
	alpha=90	beta=114.7162(17)	gamma=90
Temperature:	100 K		

	Calculated	Reported
Volume	5879.28(15)	5879.29(13)
Space group	C 2/c	C 2/c
Hall group	-C 2yc	-C 2yc
Moiety formula	2(C6 H2 N3 O7), C5 H11 N O2, 2(C3 H5 N2)	2(C6 H2 N3 O7), C5 H11 N O2, 2(C3 H5 N2)
Sum formula	C23 H25 N11 O16	C23 H25 N11 O16
Mr	711.54	711.51
Dx,g cm-3	1.608	1.620
Z	8	8
Mu (mm-1)	0.138	0.000
F000	2944.0	2944.0
F000’	2945.79	
h,k,lmax	80,18,60	79,18,60
Nref	42631	31489
Tmin,Tmax		
Tmin’		

Correction method= Not given

Data completeness= 0.739 Theta(max)= 58.563

R(reflections)= 0.0365(23751) wR2(reflections)= wR= 0.0456(23751)

S = 1.013 Npar= 676

The following ALERTS were generated. Each ALERT has the format
test-name_ALERT_alert-type_alert-level.
Click on the hyperlinks for more details of the test.

Alert level B

```
PLAT230_ALERT_2_B Hirshfeld Test Diff for    O4A    --  N5A     ..        7.7 s.u.
PLAT230_ALERT_2_B Hirshfeld Test Diff for    O3B    --  N4B     ..        8.1 s.u.
PLAT230_ALERT_2_B Hirshfeld Test Diff for    N6B    --  C11B    ..        8.0 s.u.
PLAT353_ALERT_3_B Long    N-H (N0.87,N1.01A)  N3A    -   H3A    ..       1.08 Ang.
```

Alert level C

```
PLAT230_ALERT_2_C Hirshfeld Test Diff for    O5A    --  N5A     ..        6.3 s.u.
PLAT230_ALERT_2_C Hirshfeld Test Diff for    O6A    --  N6A     ..        5.5 s.u.
PLAT230_ALERT_2_C Hirshfeld Test Diff for    O7B    --  N6B     ..        5.6 s.u.
PLAT351_ALERT_3_C Long    C-H (X0.96,N1.08A)  C15    -   H152   ..       1.11 Ang.
PLAT351_ALERT_3_C Long    C-H (X0.96,N1.08A)  C16    -   H162   ..       1.11 Ang.
PLAT353_ALERT_3_C Long    N-H (N0.87,N1.01A)  N1A    -   H1A    ..       1.06 Ang.
PLAT353_ALERT_3_C Long    N-H (N0.87,N1.01A)  N1B    -   H1B    ..       1.04 Ang.
PLAT353_ALERT_3_C Long    N-H (N0.87,N1.01A)  N3B    -   H3B    ..       1.03 Ang.
```

Alert level G

```
PLAT005_ALERT_5_G No Embedded Refinement Details found  in the CIF     Please Do !
PLAT128_ALERT_4_G Alternate Setting for Input Space Group     C2/c       I2/a Note
PLAT152_ALERT_1_G The Supplied and Calc. Volume s.u. Differ by ...          2 Units
PLAT231_ALERT_4_G Hirshfeld Test (Solvent)   N7     --  C14     ..        5.5 s.u.
PLAT432_ALERT_2_G Short Inter X...Y Contact  O1A    ..  C7B     ..       2.97 Ang.
PLAT432_ALERT_2_G Short Inter X...Y Contact  O5A    ..  C14     ..       2.93 Ang.
PLAT432_ALERT_2_G Short Inter X...Y Contact  C2B    ..  C10A    ..       3.18 Ang.
PLAT790_ALERT_4_G Centre of Gravity not Within Unit Cell: Resd.  #          3 Note
              C5 H11 N O2
PLAT808_ALERT_5_G No Parseable SHELXL Style Weighting Scheme Found     Please Check
PLAT980_ALERT_1_G No Anomalous Scattering Factors Found in CIF ...     Please Check
```

0 **ALERT level A** = Most likely a serious problem - resolve or explain
4 **ALERT level B** = A potentially serious problem, consider carefully
8 **ALERT level C** = Check. Ensure it is not caused by an omission or oversight
10 **ALERT level G** = General information/check it is not something unexpected

2 ALERT type 1 CIF construction/syntax error, inconsistent or missing data
9 ALERT type 2 Indicator that the structure model may be wrong or deficient
6 ALERT type 3 Indicator that the structure quality may be low
3 ALERT type 4 Improvement, methodology, query or suggestion
2 ALERT type 5 Informative message, check

It is advisable to attempt to resolve as many as possible of the alerts in all categories. Often the minor alerts point to easily fixed oversights, errors and omissions in your CIF or refinement strategy, so attention to these fine details can be worthwhile. In order to resolve some of the more serious problems it may be necessary to carry out additional measurements or structure refinements. However, the purpose of your study may justify the reported deviations and the more serious of these should normally be commented upon in the discussion or experimental section of a paper or in the "special_details" fields of the CIF. checkCIF was carefully designed to identify outliers and unusual parameters, but every test has its limitations and alerts that are not important in a particular case may appear. Conversely, the absence of alerts does not guarantee there are no aspects of the results needing attention. It is up to the individual to critically assess their own results and, if necessary, seek expert advice.

Publication of your CIF in IUCr journals

A basic structural check has been run on your CIF. These basic checks will be run on all CIFs submitted for publication in IUCr journals (*Acta Crystallographica*, *Journal of Applied Crystallography*, *Journal of Synchrotron Radiation*); however, if you intend to submit to *Acta Crystallographica Section C* or *E* or *IUCrData*, you should make sure that full publication checks are run on the final version of your CIF prior to submission.

Publication of your CIF in other journals

Please refer to the *Notes for Authors* of the relevant journal for any special instructions relating to CIF submission.

PLATON version of 27/03/2017; check.def file version of 24/03/2017

Datablock bipa_tzvp_c - ellipsoid plot

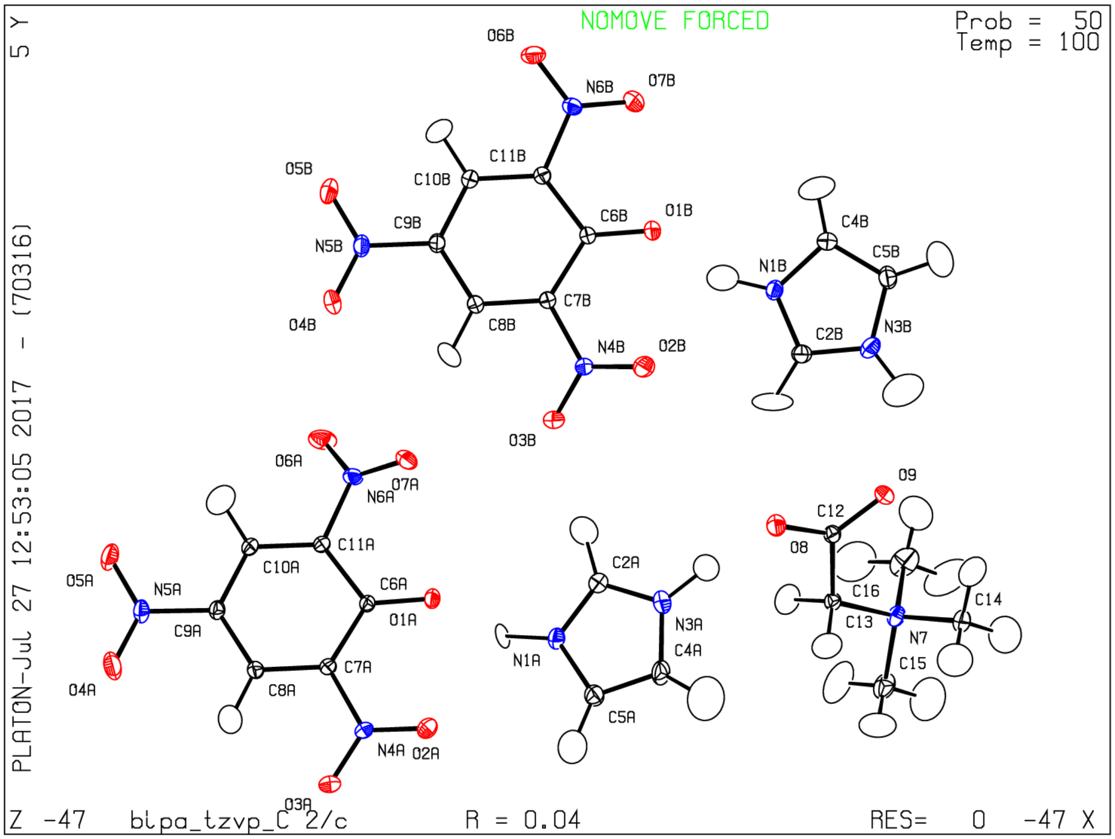

Supplement: Supplementary file 2 [file m-05-00032-sup2.zip › bipa_checkcif.pdf]
