# Supplementary material for: Probing the accuracy and precision of Hirshfeld atom refinement with HARt interfaced with Olex2
Source: IUCrJ. 2018 Jan 1;5(Pt 1):32–44. doi: 10.1107/S2052252517015548 (PMC5755575; doi:10.1107/S2052252517015548)

checkCIF/PLATON report

You have not supplied any structure factors. As a result the full set of tests cannot be run.

THIS REPORT IS FOR GUIDANCE ONLY. IF USED AS PART OF A REVIEW PROCEDURE FOR PUBLICATION, IT SHOULD NOT REPLACE THE EXPERTISE OF AN EXPERIENCED CRYSTALLOGRAPHIC REFEREE.

No syntax errors found. CIF dictionary Interpreting this report

Datablock: oxalate-tzvp-cl

```
Bond precision:  C-C = 0.0006 A          Wavelength=0.56160

Cell:             a=4.265(1)     b=12.796(1)        c=7.490(1)
                  alpha=90       beta=100.77(1)     gamma=90
Temperature:      11 K

                Calculated                 Reported
Volume          401.57(11)                 401.57(11)
Space group     P 21/c                     P 1 21/c 1
Hall group      -P 2ybc                    -P 2ybc
Moiety formula  C2 H K O4                  C2 H1 K1 O4
Sum formula     C2 H K O4                  C2 H1 K1 O4
Mr              128.13                     128.13
Dx,g cm-3       2.119                      0.000
Z               4                          4
Mu (mm-1)       0.613                      0.000
F000            256.0                      0.0
F000'           256.65
h,k,lmax
Nref
Tmin,Tmax
Tmin'

Correction method= Not given

Data completeness=                 Theta(max)=

R(reflections)= 0.0196( 0)         wR2(reflections)= wR= 0.0246(
                                   4439)

S = 1.340                 Npar= 73
```

The following ALERTS were generated. Each ALERT has the format
test-name_ALERT_alert-type_alert-level.
Click on the hyperlinks for more details of the test.

Alert level C

```
CELLK01_ALERT_1_C  Check that the cell measurement temperature is in Kelvin.
            Value of measurement temperature given =    11.000
PLAT250_ALERT_2_C Large U3/U1 Ratio for Average U(i,j) Tensor ....        2.2 Note
PLAT355_ALERT_3_C Long    O-H (X0.82,N0.98A)  O1     -   H1     ..       1.04 Ang.
```

Alert level G

```
PLAT004_ALERT_5_G Polymeric Structure Found with Maximum Dimension          3 Info
PLAT005_ALERT_5_G No Embedded Refinement Details found  in the CIF     Please Do !
PLAT092_ALERT_4_G Check: Wavelength given is not Cu,Ga,Mo,Ag,In Ka    0.56160 Ang.
PLAT153_ALERT_1_G The s.u.'s on the Cell Axes   are Equal ..(Note)      0.001 Ang.
PLAT780_ALERT_1_G Coordinates do not Form a Properly Connected Set     Please Do !
PLAT804_ALERT_5_G Number of ARU-Code Packing Problem(s) in PLATON          82 Info
PLAT808_ALERT_5_G No Parseable SHELXL Style Weighting Scheme Found     Please Check
PLAT881_ALERT_1_G Missing datum for _diffrn_reflns_av_R_equivalents    Please Check
PLAT882_ALERT_1_G Missing datum for _diffrn_reflns_av_unetI/netI .     Please Check
PLAT980_ALERT_1_G No Anomalous Scattering Factors Found in CIF ...     Please Check
```

   0 **ALERT level A** = Most likely a serious problem - resolve or explain
   0 **ALERT level B** = A potentially serious problem, consider carefully
   3 **ALERT level C** = Check. Ensure it is not caused by an omission or oversight
  10 **ALERT level G** = General information/check it is not something unexpected

   6 ALERT type 1 CIF construction/syntax error, inconsistent or missing data
   1 ALERT type 2 Indicator that the structure model may be wrong or deficient
   1 ALERT type 3 Indicator that the structure quality may be low
   1 ALERT type 4 Improvement, methodology, query or suggestion
   4 ALERT type 5 Informative message, check

It is advisable to attempt to resolve as many as possible of the alerts in all categories. Often the minor alerts point to easily fixed oversights, errors and omissions in your CIF or refinement strategy, so attention to these fine details can be worthwhile. In order to resolve some of the more serious problems it may be necessary to carry out additional measurements or structure refinements. However, the purpose of your study may justify the reported deviations and the more serious of these should normally be commented upon in the discussion or experimental section of a paper or in the "special_details" fields of the CIF. checkCIF was carefully designed to identify outliers and unusual parameters, but every test has its limitations and alerts that are not important in a particular case may appear. Conversely, the absence of alerts does not guarantee there are no aspects of the results needing attention. It is up to the individual to critically assess their own results and, if necessary, seek expert advice.

Publication of your CIF in IUCr journals

A basic structural check has been run on your CIF. These basic checks will be run on all CIFs submitted for publication in IUCr journals (*Acta Crystallographica*, *Journal of Applied Crystallography*, *Journal of Synchrotron Radiation*); however, if you intend to submit to *Acta Crystallographica Section C* or *E* or *IUCrData*, you should make sure that full publication checks are run on the final version of your CIF prior to submission.

Publication of your CIF in other journals

Please refer to the *Notes for Authors* of the relevant journal for any special instructions relating to CIF submission.

PLATON version of 27/03/2017; check.def file version of 24/03/2017

Datablock oxalate-tzvp-cl - ellipsoid plot

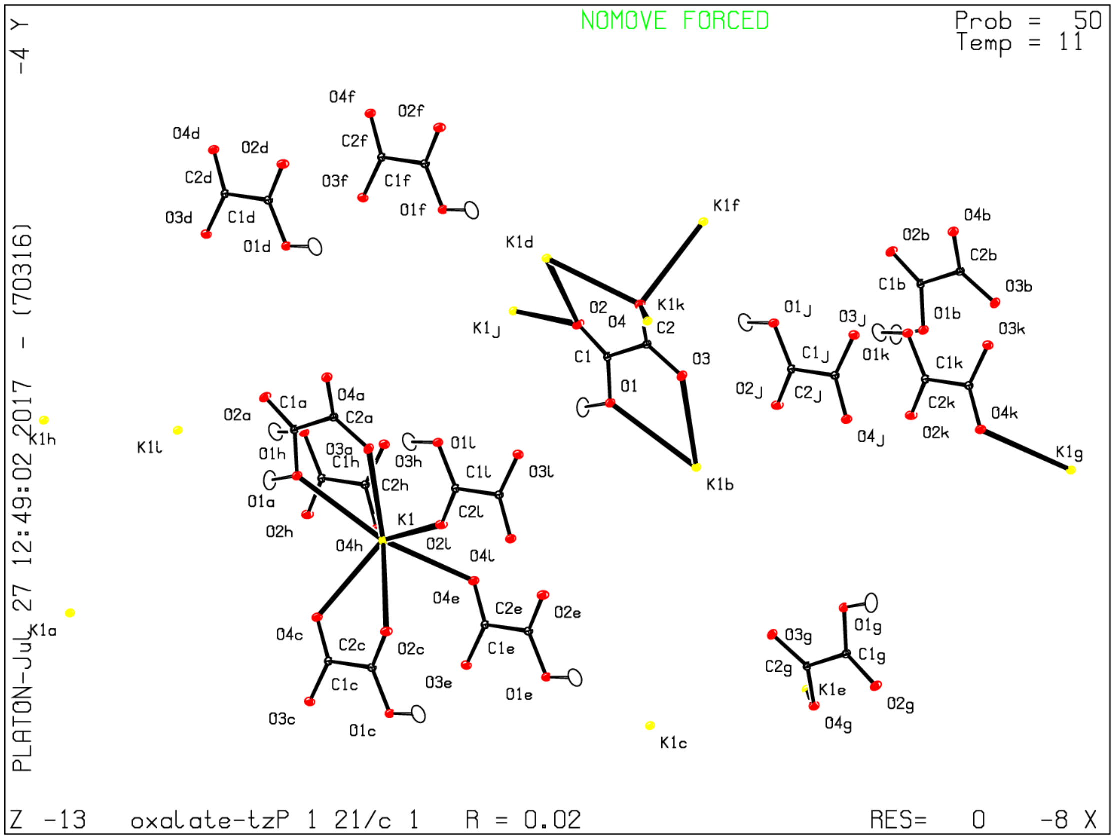

Supplement: Supplementary file 4 [file m-05-00032-sup4.zip › oxalate_checkcif.pdf]
