# Supplementary material for: Probing the accuracy and precision of Hirshfeld atom refinement with HARt interfaced with Olex2
Source: IUCrJ. 2018 Jan 1;5(Pt 1):32–44. doi: 10.1107/S2052252517015548 (PMC5755575; doi:10.1107/S2052252517015548)
Supplement: Supplementary file 5 [file m-05-00032-sup5.pdf]

IUCrJ

Volume 5 (2018)

Supporting information for article:

Probing accuracy and precision of Hirshfeld Atom Refinement with HARt interfaced to Olex2

Malte Fugel, Dylan Jayatilaka, Emanuel Hupf, Jacob Overgaard, Venkatesha R. Hathwar, Piero Macchi, Michael J. Turner, Judith A. K. Howard, Oleg V. Dolomanov, Horst Puschmann, Bo B. Iversen, Hans-Beat Bürgi and Simon Grabowsky

Supporting information for:
Probing accuracy and precision of Hirshfeld Atom Refinement with HARt interfaced to Olex2

Malte Fugel[a], Dylan Jayatilaka[b], Emanuel Hupf[a], Jacob Overgaard[c], Venkatesha R. Hathwar[c,d], Piero Macchi[e], Michael J. Turner[b], Judith A. K. Howard[f], Oleg V. Dolomanov[f], Horst Puschmann[f], Bo B. Iversen[c], Hans-Beat Bürgi[e,g], and Simon Grabowsky[a*]

[a]Department 2: Biology/Chemistry, University of Bremen, Leobener Str. NW2, 28359 Bremen, Germany
[b]School of Chemistry and Biochemistry, University of Western Australia, 35 Stirling Highway, 6009 Perth, Australia
[c]Center for Materials Crystallography, Department of Chemistry and iNano, Aarhus University, Langelandsgade 140, 8000 Aarhus, Denmark
[d]Division of Physics, Faculty of Pure and Applied Sciences, University of Tsukuba, 305-8571, Tsukuba, Japan
[e]Department of Chemistry and Biochemistry, University of Bern, Freiestraße 3, 3012 Bern, Switzerland
[f]Department of Chemistry, Durham University, South Road, Durham DH1 3LE, UK
[g]Department of Chemistry, University of Zürich, Winterthurerstraße 190, 8057 Zürich, Switzerland

1 Residual density representations in different molecular planes for BIPa

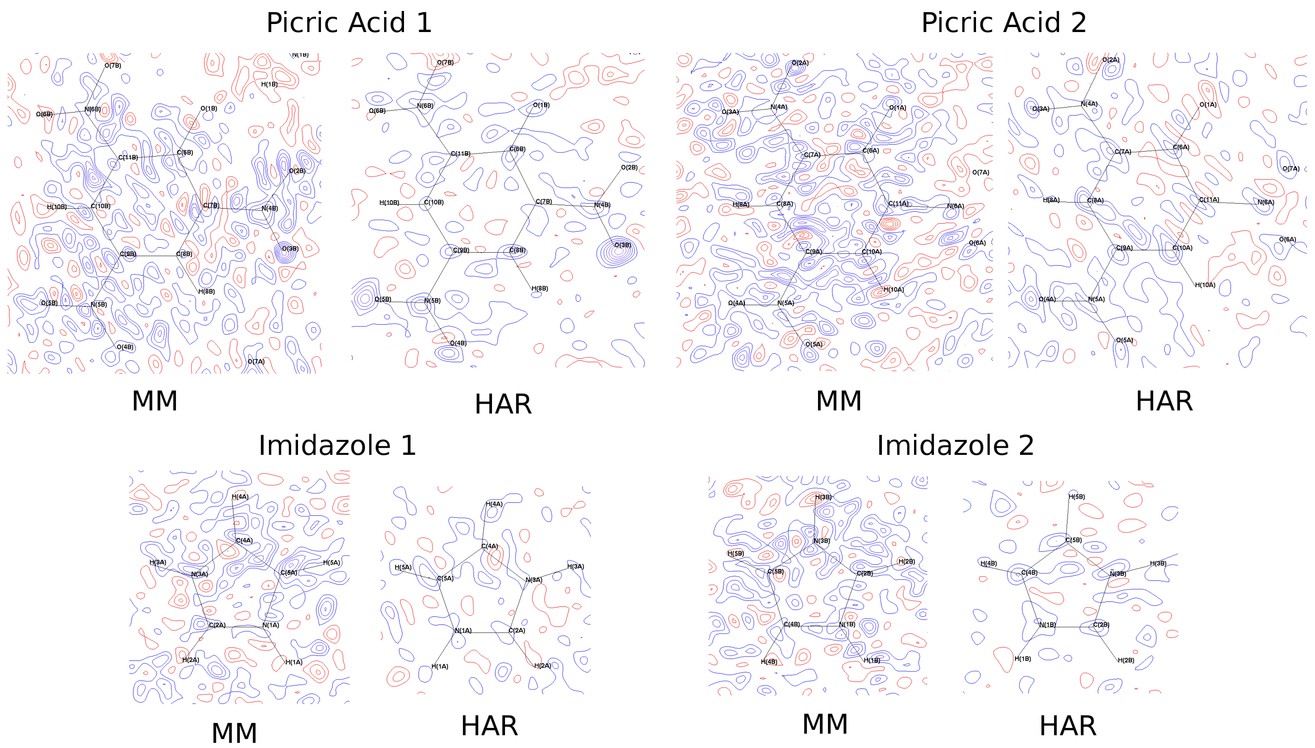

Figure S1. Residual density maps in the ring planes of BIPa for the MM/SHADE model and HAR (def2-TZVP, charges), calculated with the full resolution as given in Table 2, contour intervals = 0.05 e$Å^{-3}$, blue = positive, red = negative

2 Discussion of a multipole model with hydrogen ADPs from the neutron data for rubrene

One may argue that the accuracy and precision of the non-hydrogen ADPs in MMs are affected by the origin of the hydrogen ADPs. Here we show that the measures of accuracy and precision for the non-hydrogen ADPs are essentially the same whether the H-ADPs are obtained from the SHADE procedure or from a neutron diffraction experiment (Table S1, bottom). The respective residual density maps show no significant differences; the same features appear in both (Figure S2). It can thus be concluded that comparisons of non-hydrogen ADPs with those of MM/SHADE models are not biased by the choice of H-ADPs.

Table S1. Anisotropic displacement parameters for non-hydrogen atoms and parameters of accuracy and precision of the MM with the hydrogen ADPs fixed to values from neutron diffraction, units Å^2

	U11	U22	U33	U12	U13	U23
C(1)	0.00994(8)	0.00904(9)	0.01080(9)	0	0	0.00129(7)
C(2)	0.01055(6)	0.00976(7)	0.01102(7)	0.00073(5)	-0.00006(5)	0.00127(5)
C(3)	0.01219(7)	0.00929(7)	0.01055(7)	0.00081(5)	0.00020(5)	0.00135(5)
C(4)	0.01503(8)	0.01164(8)	0.01386(8)	0.00274(6)	0.00110(6)	0.00330(6)
C(5)	0.01872(9)	0.01132(8)	0.01539(8)	0.00205(6)	0.00073(6)	0.00397(6)
C(6)	0.01084(7)	0.01185(7)	0.01171(7)	0.00132(5)	-0.00026(5)	0.00051(5)
C(7)	0.01225(7)	0.01440(8)	0.01759(9)	0.00173(6)	0.00201(6)	-0.00103(7)
C(8)	0.01156(8)	0.02023(11)	0.02494(11)	0.00268(7)	0.00224(7)	0.00167(9)
C(9)	0.01259(9)	0.02714(13)	0.02326(12)	0.00033(8)	-0.00323(8)	0.00373(10)
C(10)	0.01672(10)	0.02707(13)	0.01694(10)	-0.00134(9)	-0.00507(7)	-0.00140(9)
C(11)	0.01461(8)	0.01874(9)	0.01328(8)	0.00127(7)	-0.00176(6)	-0.00238(7)
	$\langle U_X^{ii}/U_N^{ii}\rangle$	$\lvert\Delta U_{X-N}^{ij}\rvert$	$\lvert\Delta U_{X-N}^{ii}\rvert$	wRMSD		
MM/neutron	1.01(3)	0.00021(18)	0.00026(20)	1.81		
MM/SHADE	0.99(3)	0.00021(17)	0.00026(19)	1.84		

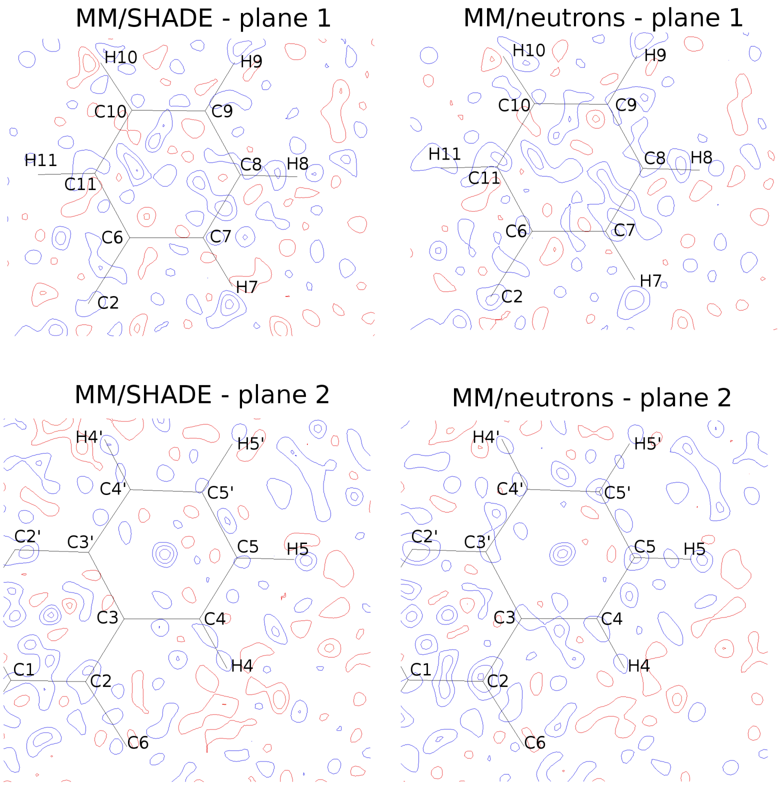

Figure S2. Residual density maps of two planes of rubrene from the MMs with the hydrogen ADPs obtained from neutron diffraction and the SHADE approach, alternatively; contour intervals = 0.05 eÅ^{-3}, blue = positive, red = negative, full resolution as given in Table 2

3 Individual anisotropic displacement parameters

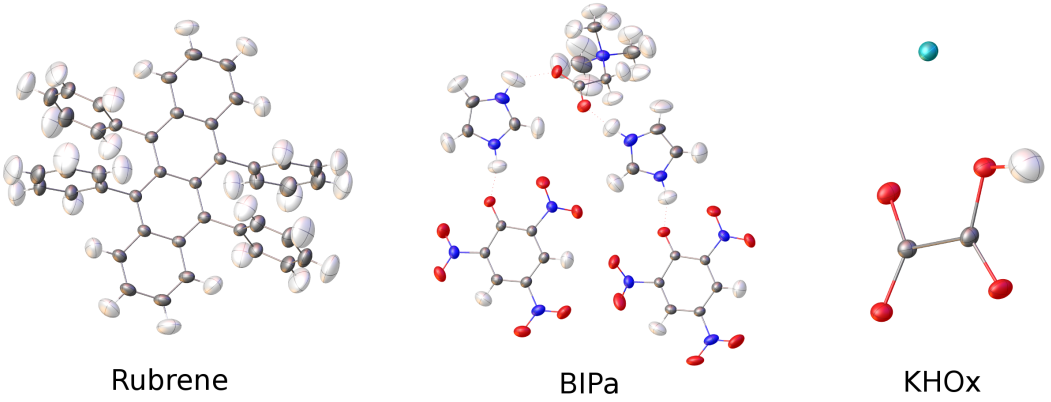

Figure S3. Anisotropic displacement parameters (90% probability surface) obtained from the refinement of the neutron diffraction data of rubrene (left), BIPa (middle) and KHOx (right)

Table S2. Anisotropic displacement parameters of the neutron data of rubrene, units = Å^2

	U11	U22	U33	U12	U13	U23
C1	0.00972(14)	0.00961(16)	0.01000(12)	0	0	0.00128(10)
C2	0.01055(10)	0.00988(11)	0.01081(9)	0.00085(8)	0.00014(7)	0.00104(7)
C3	0.01197(10)	0.00962(11)	0.01017(9)	0.00081(8)	0.00030(7)	0.00145(7)
C4	0.01527(12)	0.01163(12)	0.01362(10)	0.00258(9)	0.00125(8)	0.00323(8)
C5	0.01890(13)	0.01151(13)	0.01519(11)	0.00195(10)	0.00095(9)	0.00431(9)
C6	0.01057(10)	0.01180(12)	0.01127(9)	0.00134(8)	-0.00027(7)	0.00068(8)
C7	0.01196(11)	0.01435(14)	0.01764(11)	0.00161(9)	0.00217(9)	-0.00129(9)
C8	0.01101(12)	0.02017(16)	0.02509(14)	0.00269(10)	0.00259(10)	0.00194(11)
C9	0.01212(13)	0.02789(19)	0.02328(13)	-0.00009(11)	-0.00322(10)	0.00430(12)
C10	0.01691(14)	0.02736(19)	0.01664(11)	-0.00163(12)	-0.00526(10)	-0.00124(11)
C11	0.01433(13)	0.01907(15)	0.01303(10)	0.00120(10)	-0.00179(9)	-0.00265(9)
H4	0.0205(3)	0.0317(4)	0.0400(3)	0.0049(3)	0.0018(3)	0.0113(3)
H5	0.0346(4)	0.0264(4)	0.0415(4)	0.0077(3)	0.0003(3)	0.0152(3)
H7	0.0301(4)	0.0336(4)	0.0349(3)	0.0016(3)	0.00100(3)	-0.0146(3)
H8	0.0239(4)	0.0387(5)	0.0560(5)	0.0087(3)	0.00800(3)	-0.0074(4)
H9	0.0201(4)	0.0592(6)	0.0495(5)	0.0011(3)	-0.00970(3)	0.0017(4)
H10	0.0379(5)	0.0575(6)	0.0343(4)	-0.0046(4)	-0.0126(3)	-0.0157(4)
H11	0.0297(4)	0.0417(5)	0.0342(3)	0.0082(3)	0.0004(3)	-0.0156(3)

Table S3. Anisotropic displacement parameters of the independent atom model of rubrene, units = Å^2

	U11	U22	U33	U12	U13	U23
C1	0.01014(13)	0.00929(13)	0.01065(13)	0	0	0.00102(10)
C2	0.01080(10)	0.00999(10)	0.01095(10)	0.00082(7)	-0.00002(7)	0.00112(7)
C3	0.01246(11)	0.00946(10)	0.01041(10)	0.00084(7)	0.00021(7)	0.00113(7)
C4	0.01538(12)	0.01186(11)	0.01366(11)	0.00291(9)	0.00094(9)	0.00294(9)
C5	0.01903(14)	0.01152(11)	0.01520(13)	0.00216(9)	0.00071(10)	0.00363(9)
C6	0.01105(10)	0.01185(10)	0.01183(10)	0.00121(8)	-0.00015(8)	0.00087(8)
C7	0.01257(11)	0.01445(12)	0.01752(13)	0.00167(9)	0.00216(9)	-0.00068(10)
C8	0.01188(12)	0.02010(16)	0.02504(17)	0.00266(11)	0.00250(11)	0.00220(13)
C9	0.01262(13)	0.0273(2)	0.02365(18)	0.00001(12)	-0.00312(11)	0.00449(15)
C10	0.01724(14)	0.0271(2)	0.01695(15)	-0.00160(13)	-0.00491(11)	-0.00100(13)
C11	0.01496(12)	0.01863(14)	0.01332(12)	0.00102(10)	-0.00164(9)	-0.00200(10)

Table S4. Anisotropic displacement parameters of the MM of rubrene (hydrogen ADPs are obtained from SHADE), units = Å^2

	U11	U22	U33	U12	U13	U23
C1	0.00998(9)	0.00910(9)	0.01081(10)	0	0	0.00125(8)
C2	0.01054(7)	0.00972(7)	0.01108(7)	0.00075(5)	-0.00007(5)	0.00129(6)
C3	0.01219(8)	0.00926(7)	0.01055(7)	0.00080(5)	0.00019(5)	0.00134(6)
C4	0.01505(9)	0.01165(8)	0.01380(8)	0.00274(6)	0.00108(6)	0.00331(6)
C5	0.01874(10)	0.01127(8)	0.01533(9)	0.00204(7)	0.00075(7)	0.00393(7)
C6	0.01083(7)	0.01184(8)	0.01174(7)	0.00135(6)	-0.00026(6)	0.00060(6)
C7	0.01219(8)	0.01442(9)	0.01763(9)	0.00173(7)	0.00203(7)	-0.00101(7)
C8	0.01155(9)	0.02026(12)	0.02488(12)	0.00272(8)	0.00222(8)	0.00168(10)
C9	0.01243(9)	0.02714(14)	0.02339(13)	0.00033(9)	-0.00325(8)	0.00378(11)
C10	0.01675(10)	0.02705(14)	0.01691(11)	-0.00130(10)	-0.00506(8)	-0.00137(9)
C11	0.01449(9)	0.01874(10)	0.01328(9)	0.00124(7)	-0.00175(7)	-0.00236(7)
H4	0.020528	0.029586	0.036105	0.006668	-0.001335	0.003129
H5	0.035014	0.024093	0.038192	0.012973	-0.003162	0.006032
H7	0.026733	0.035666	0.03504	-0.014106	0.002014	-0.000367
H8	0.022902	0.038456	0.052812	-0.007858	0.005226	0.008128
H9	0.018521	0.051708	0.043601	0.002324	-0.008475	0.002817
H10	0.029899	0.056191	0.032861	-0.015203	-0.008727	-0.00233
H11	0.023989	0.038364	0.034177	-0.013843	-0.001371	0.004924

Table S5. Anisotropic displacement parameters of the HAR (HF/def2-SVP) of rubrene, units = Å^2

	U11	U22	U33	U12	U13	U23
C1	0.00993(8)	0.00908(9)	0.01042(9)	0	0	0.00123(7)
C2	0.01044(6)	0.00967(6)	0.01079(6)	0.00073(5)	-0.00005(5)	0.00129(5)
C3	0.01212(7)	0.00920(7)	0.01027(7)	0.00085(5)	0.00022(5)	0.00137(5)
C4	0.01493(7)	0.01150(7)	0.01359(7)	0.00266(6)	0.00107(6)	0.00329(6)
C5	0.01867(9)	0.01115(8)	0.01511(8)	0.00206(6)	0.00077(6)	0.00398(6)
C6	0.01079(6)	0.01172(7)	0.01145(7)	0.00133(5)	-0.00023(5)	0.00048(5)
C7	0.01219(7)	0.01432(8)	0.01727(8)	0.00168(6)	0.00207(6)	-0.00110(6)
C8	0.01146(7)	0.0201(1)	0.0246(1)	0.00278(7)	0.00226(7)	0.00165(8)
C9	0.01230(8)	0.0272(1)	0.0230(1)	0.00016(8)	-0.00329(7)	0.00385(9)
C10	0.01677(9)	0.0270(1)	0.01668(9)	-0.00136(8)	-0.00499(7)	-0.00139(8)
C11	0.01442(8)	0.01874(9)	0.01306(8)	0.00130(6)	-0.00178(6)	-0.00246(6)
H4	0.028(3)	0.025(3)	0.035(3)	0.007(2)	0.002(2)	0.016(3)
H5	0.032(3)	0.033(3)	0.045(4)	0.008(3)	0.002(3)	0.016(3)
H7	0.031(3)	0.041(4)	0.041(4)	-0.006(3)	0.007(3)	-0.004(3)
H8	0.022(3)	0.048(4)	0.059(4)	0.004(3)	0.008(3)	-0.006(4)
H9	0.032(3)	0.064(5)	0.042(4)	-0.002(3)	-0.005(3)	0.000(4)
H10	0.043(4)	0.057(5)	0.033(4)	-0.012(3)	-0.011(3)	-0.010(3)
H11	0.025(3)	0.048(4)	0.034(3)	0.004(3)	-0.008(3)	-0.005(3)

Table S6. Anisotropic displacement parameters of the HAR (HF/def2-TZVP) of rubrene, units = Å^2

	U11	U22	U33	U12	U13	U23
C1	0.00988(8)	0.00902(9)	0.01038(9)	0	0	0.00126(7)
C2	0.01039(6)	0.00961(6)	0.01076(6)	0.00072(5)	-0.00006(5)	0.00132(5)
C3	0.01205(7)	0.00914(6)	0.01024(7)	0.00085(5)	0.00022(5)	0.00139(5)
C4	0.01485(7)	0.01145(7)	0.01358(7)	0.00264(6)	0.00107(6)	0.00334(6)
C5	0.01860(9)	0.01109(8)	0.01509(8)	0.00204(6)	0.00079(6)	0.00403(6)
C6	0.01074(6)	0.01167(7)	0.01139(7)	0.00135(5)	-0.00024(5)	0.00041(5)
C7	0.01212(7)	0.01430(8)	0.01723(8)	0.00170(6)	0.00205(6)	-0.00116(6)
C8	0.01138(7)	0.0201(1)	0.0245(1)	0.00279(7)	0.00223(7)	0.00156(8)
C9	0.01223(8)	0.0272(1)	0.0229(1)	0.00019(8)	-0.00332(7)	0.00375(9)
C10	0.01666(9)	0.0269(1)	0.01662(9)	-0.00133(8)	-0.00499(7)	-0.00145(8)
C11	0.01434(8)	0.01871(9)	0.01302(8)	0.00132(6)	-0.00181(6)	-0.00252(6)
H4	0.027(3)	0.027(3)	0.038(3)	0.008(2)	0.001(2)	0.016(3)
H5	0.031(3)	0.036(3)	0.049(4)	0.010(3)	-0.000(3)	0.018(3)
H7	0.035(3)	0.042(4)	0.042(4)	-0.007(3)	0.006(3)	-0.008(3)
H8	0.023(3)	0.051(4)	0.061(4)	0.007(3)	0.008(3)	-0.007(4)
H9	0.032(3)	0.064(5)	0.044(4)	-0.001(3)	-0.006(3)	0.000(4)
H10	0.045(4)	0.061(5)	0.034(4)	-0.014(4)	-0.012(3)	-0.012(3)
H11	0.026(3)	0.048(4)	0.037(3)	0.004(3)	-0.006(3)	-0.007(3)

Table S7. Anisotropic displacement parameters of the HAR (HF/def2-TZVP, with charges) of rubrene, units = Å^2

	U11	U22	U33	U12	U13	U23
C1	0.00987(8)	0.00900(8)	0.01044(9)	0	0	0.00126(7)
C2	0.01038(6)	0.00960(6)	0.01082(6)	0.00072(5)	-0.00007(5)	0.00133(5)
C3	0.01205(7)	0.00913(6)	0.01030(6)	0.00075(5)	0.00018(5)	0.0014(5)
C4	0.01485(7)	0.01144(7)	0.01364(7)	0.00264(6)	0.00106(6)	0.00334(6)
C5	0.01859(9)	0.01108(8)	0.01519(8)	0.00206(6)	0.00079(6)	0.00406(6)
C6	0.01072(6)	0.01166(7)	0.01147(7)	0.00136(5)	-0.00024(5)	0.0004(5)
C7	0.01210(7)	0.01429(8)	0.01733(8)	0.00171(6)	0.00206(6)	-0.00117(6)
C8	0.01137(7)	0.0201(1)	0.0247(1)	0.00279(7)	0.00223(7)	0.00158(8)
C9	0.01221(9)	0.0272(1)	0.0230(1)	0.00020(8)	-0.00334(8)	0.00376(8)
C10	0.01665(8)	0.0269(1)	0.01669(9)	-0.00133(8)	-0.005(6)	-0.00148(6)
C11	0.01432(8)	0.01871(9)	0.01309(8)	0.00134(6)	-0.00181(8)	-0.00255(9)
H4	0.027(3)	0.027(3)	0.039(3)	0.008(2)	0.002(2)	0.016(3)
H5	0.031(3)	0.035(3)	0.049(4)	0.01(3)	0.000(3)	0.018(3)
H7	0.034(3)	0.042(3)	0.042(4)	-0.007(3)	0.006(3)	-0.007(3)
H8	0.023(3)	0.051(4)	0.061(4)	0.007(3)	0.008(3)	-0.006(4)
H9	0.032(4)	0.063(5)	0.044(4)	-0.001(3)	-0.006(3)	0.000(3)
H10	0.045(3)	0.059(4)	0.034(3)	-0.015(3)	-0.012(3)	-0.012(3)
H11	0.026(3)	0.048(5)	0.037(4)	0.004(3)	-0.006(3)	-0.008(4)

Table S8. Non-hydrogen anisotropic displacement parameters of the neutron data of BIPa, units = Å^2

	U11	U22	U33	U12	U13	U23
O8	0.0127(3)	0.0111(3)	0.0192(3)	-0.0005(3)	0.0050(3)	0.0005(3)
O9	0.0104(3)	0.0195(4)	0.0204(4)	0.0002(3)	0.0076(3)	0.0040(3)
N7	0.0144(2)	0.01136(18)	0.0114(2)	0.00137(16)	0.0035(2)	-0.00007(16)
C12	0.0092(2)	0.0118(2)	0.0103(2)	-0.0010(2)	0.0033(2)	0.0007(2)
C13	0.0093(2)	0.0113(3)	0.0127(3)	-0.00009(19)	0.0040(2)	0.0013(2)
C14	0.0136(3)	0.0181(3)	0.0153(3)	0.0038(3)	0.0033(2)	0.0038(3)
C15	0.0187(3)	0.0138(3)	0.0273(4)	-0.0033(3)	0.0030(3)	0.0048(3)
C16	0.0445(6)	0.0214(4)	0.0166(3)	0.0061(4)	0.0136(4)	-0.0051(3)
N1A	0.0119(2)	0.0153(2)	0.01235(19)	0.00197(16)	0.0028(2)	0.00096(16)
N3A	0.0115(2)	0.0160(2)	0.0175(2)	0.00161(16)	0.0043(2)	-0.00093(18)
C2A	0.0154(3)	0.0145(3)	0.0152(3)	0.0013(2)	0.0068(2)	0.0023(2)
C4A	0.0155(3)	0.0243(4)	0.0113(3)	0.0057(3)	0.0022(2)	0.0020(3)
C5A	0.0146(3)	0.0243(3)	0.0133(3)	0.0044(3)	0.0060(2)	0.0028(3)
O1A	0.0124(3)	0.0175(3)	0.0121(3)	0.0054(3)	0.0050(3)	0.0036(3)
O2A	0.0171(4)	0.0349(5)	0.0144(3)	0.0070(4)	0.0063(3)	0.0114(4)
O3A	0.0204(4)	0.0244(4)	0.0180(4)	0.0029(3)	0.0132(3)	0.0073(3)
O4A	0.0161(4)	0.0377(5)	0.0236(4)	0.0131(4)	0.0084(3)	0.0040(4)
O5A	0.0185(4)	0.0228(4)	0.0169(4)	0.0084(3)	0.0021(3)	0.0071(3)
O6A	0.0221(4)	0.0380(5)	0.0126(3)	-0.0045(4)	0.0108(3)	-0.0014(4)
O7A	0.0121(3)	0.0307(4)	0.0197(4)	0.00266(3)	0.0089(3)	0.0051(4)
N4A	0.0145(2)	0.01537(19)	0.01119(18)	0.00113(17)	0.0066(2)	0.0032(2)
N5A	0.0114(2)	0.0169(2)	0.0142(2)	0.00415(16)	0.0015(2)	0.00013(17)
N6A	0.0134(2)	0.0164(2)	0.01228(18)	-0.00020(16)	0.0076(2)	0.00050(16)
C6A	0.0090(2)	0.0110(2)	0.0083(2)	0.00128(19)	0.00339(19)	0.00058(19)
C7A	0.0099(2)	0.0109(2)	0.0092(2)	0.00084(19)	0.0042(2)	0.0015(2)
C8A	0.0094(3)	0.0129(3)	0.0110(3)	0.0010(2)	0.0043(2)	0.0007(2)
C9A	0.0096(2)	0.0121(2)	0.0104(2)	0.0018(2)	0.0030(2)	0.0010(2)
C10A	0.0111(3)	0.0125(3)	0.0092(2)	0.0010(2)	0.0032(2)	0.0019(2)
C11A	0.0100(2)	0.0115(2)	0.0086(2)	0.0007(2)	0.00415(19)	0.0010(2)
N1B	0.0121(2)	0.01323(19)	0.01258(19)	0.0012(2)	0.00415(15)	0.00189(16)
N3B	0.0198(2)	0.0151(2)	0.01242(19)	0.0034(2)	0.00597(17)	0.00289(17)
C2B	0.0148(3)	0.0132(3)	0.0154(2)	-0.0002(2)	0.0084(2)	0.0005(2)
C4B	0.0151(3)	0.0173(3)	0.0159(3)	0.0013(2)	0.0090(2)	0.0024(3)
C5B	0.0124(3)	0.0207(3)	0.0176(3)	0.0045(2)	0.0051(2)	0.0016(3)
O1B	0.0108(3)	0.0217(4)	0.0154(3)	0.0047(3)	0.0060(3)	0.0063(3)
O2B	0.0149(4)	0.0343(5)	0.0127(3)	0.0024(3)	0.0043(3)	0.0068(3)
O3B	0.0169(4)	0.0278(4)	0.0246(4)	0.0026(3)	0.0124(3)	0.0120(4)
O4B	0.0131(4)	0.0274(4)	0.0295(5)	0.0074(3)	0.0093(3)	0.0086(4)
O5B	0.0210(4)	0.0241(4)	0.0204(4)	0.0086(3)	0.0065(3)	0.0086(4)
O6B	0.0235(4)	0.0179(4)	0.0191(4)	-0.0001(3)	0.0145(3)	0.0030(3)
O7B	0.0222(4)	0.0322(5)	0.0270(4)	0.0112(4)	0.0177(4)	0.0095(4)
N4B	0.0129(2)	0.01375(19)	0.0120(2)	0.0014(2)	0.00647(16)	0.00173(16)
N5B	0.0114(2)	0.0142(2)	0.0176(2)	0.0029(2)	0.00364(17)	0.00243(17)
N6B	0.01333(19)	0.0158(2)	0.01299(18)	-0.0007(2)	0.00753(16)	-0.00028(17)
C6B	0.0089(2)	0.0115(2)	0.0113(3)	0.0006(2)	0.0045(2)	0.0008(2)
C7B	0.0099(2)	0.0108(2)	0.0117(3)	0.0006(2)	0.0050(2)	0.0007(2)
C8B	0.0099(3)	0.0115(3)	0.0143(3)	0.0009(2)	0.0056(2)	0.0013(2)
C9B	0.0098(3)	0.0113(3)	0.0139(3)	0.0009(2)	0.0043(2)	0.0010(2)
C10B	0.0118(3)	0.0121(3)	0.01200(3)	0.0005(2)	0.0046(2)	0.0015(2)
C11B	0.0010(2)	0.0115(2)	0.0115(2)	0.0003(2)	0.0051(2)	0.0005(2)

Table S9. Hydrogen anisotropic displacement parameters of the neutron data of BIPa, units = Å^2

	U11	U22	U33	U12	U13	U23
H131	0.0187(7)	0.0274(7)	0.0314(8)	-0.0018(6)	-0.0025(6)	0.0052(6)
H132	0.0383(9)	0.0253(7)	0.0328(8)	0.0021(6)	0.0255(7)	0.0014(6)
H141	0.0376(10)	0.0271(8)	0.0488(12)	0.0147(8)	0.0080(9)	0.0094(8)
H142	0.0211(8)	0.0437(10)	0.0420(10)	-0.0044(7)	0.0087(7)	0.0090(9)
H143	0.0362(10)	0.0566(12)	0.0184(7)	0.0048(9)	0.0088(7)	-0.0006(8)
H151	0.0267(9)	0.0362(9)	0.0503(12)	-0.0085(8)	-0.0060(8)	0.0095(9)
H152	0.0388(10)	0.0185(7)	0.0583(13)	-0.0004(7)	0.0102(9)	0.0059(8)
H153	0.0454(12)	0.0399(10)	0.0499(12)	-0.0070(9)	0.0283(10)	0.0074(9)
H161	0.0622(15)	0.0510(12)	0.0438(12)	0.0040(11)	0.0375(11)	-0.0011(10)
H162	0.1052(22)	0.0301(10)	0.0536(14)	0.0202(12)	0.0427(15)	-0.0056(10)
H163	0.0682(16)	0.0588(14)	0.0220(8)	-0.0002(12)	0.0058(9)	-0.0082(9)
H1A	0.0213(7)	0.0290(7)	0.0259(7)	0.0048(6)	0.0042(6)	0.0035(6)
H2A	0.0404(10)	0.0430(10)	0.0319(9)	0.0044(8)	0.0217(8)	0.0133(8)
H3A	0.0200(7)	0.0287(7)	0.0389(9)	0.0015(6)	0.0119(6)	0.0002(7)
H4A	0.0360(10)	0.0623(13)	0.0206(8)	0.0179(9)	0.0042(7)	0.0140(8)
H5A	0.0335(10)	0.0622(13)	0.0359(9)	0.0076(9)	0.0232(8)	0.0077(10)
H8A	0.0228(7)	0.0364(8)	0.0292(8)	0.0040(6)	0.0166(6)	0.0035(7)
H10A	0.0321(8)	0.0326(8)	0.0195(6)	0.0053(7)	0.0108(6)	0.0111(6)
H1B	0.0221(7)	0.0275(7)	0.0247(7)	0.0057(6)	0.0037(6)	0.0058(6)
H2B	0.0296(9)	0.0374(9)	0.0453(10)	-0.0027(7)	0.0259(8)	0.0027(8)
H3B	0.0589(13)	0.0306(9)	0.0245(8)	0.0096(8)	0.0170(8)	0.0103(7)
H4B	0.0410(10)	0.0422(10)	0.0423(10)	0.0033(8)	0.0294(9)	0.0154(9)
H5B	0.0218(8)	0.0511(11)	0.0468(11)	0.0123(8)	0.0098(8)	0.0088(10)
H8B	0.0223(7)	0.0311(8)	0.0324(8)	0.0037(6)	0.0177(6)	0.0055(7)
H10B	0.0307(8)	0.0330(8)	0.0260(7)	0.0051(7)	0.0138(6)	0.0124(7)

Table S10. Anisotropic displacement parameters of the IAM of BIPa, units = Å^2

	U11	U22	U33	U12	U13	U23
O8	0.01445(15)	0.01241(12)	0.01920(15)	-0.00035(10)	0.00510(12)	0.00042(10)
O9	0.01104(15)	0.02086(16)	0.01971(15)	0.00021(11)	0.00674(12)	0.00339(11)
N7	0.01536(17)	0.01205(13)	0.01155(13)	0.00090(11)	0.00370(12)	-0.00022(10)
C12	0.01048(17)	0.01350(14)	0.01036(13)	-0.00108(11)	0.00288(12)	0.00047(10)
C13	0.01131(17)	0.01170(14)	0.01332(15)	0.00014(11)	0.00400(12)	0.00135(11)
C14	0.0142(2)	0.01906(19)	0.01538(17)	0.00363(14)	0.00299(15)	0.00399(13)
C15	0.0190(2)	0.01482(18)	0.0277(3)	-0.00394(15)	0.00340(19)	0.00463(16)
C16	0.0446(4)	0.0221(2)	0.0175(2)	0.0057(2)	0.0148(2)	-0.00436(17)
N1A	0.01276(16)	0.01575(14)	0.01261(13)	0.00193(11)	0.00295(12)	0.00114(11)
N3A	0.01283(17)	0.01613(15)	0.01794(16)	0.00172(12)	0.00442(13)	-0.00108(12)
C2A	0.0170(2)	0.01481(16)	0.01628(17)	0.00147(13)	0.00694(15)	0.00188(13)
C4A	0.0167(2)	0.0245(2)	0.01183(16)	0.00486(16)	0.00260(14)	0.00127(14)
C5A	0.0164(2)	0.0243(2)	0.01349(17)	0.00381(16)	0.00630(15)	0.00221(14)
O1A	0.01315(14)	0.01756(13)	0.01276(12)	0.00544(10)	0.00440(11)	0.00309(10)
O2A	0.01776(18)	0.0349(2)	0.01456(14)	0.00640(15)	0.00520(13)	0.01070(14)
O3A	0.02067(19)	0.02442(18)	0.01942(16)	0.00224(13)	0.01332(14)	0.00626(12)
O4A	0.0178(2)	0.0386(2)	0.02345(19)	0.01243(17)	0.00870(15)	0.00346(17)
O5A	0.02136(19)	0.02355(17)	0.01638(15)	0.00678(14)	0.00215(13)	0.00673(12)
O6A	0.0249(2)	0.0369(2)	0.01223(14)	-0.00407(16)	0.01057(14)	-0.00134(13)
O7A	0.01305(17)	0.0298(2)	0.02114(17)	0.00198(13)	0.00858(13)	0.00491(14)
N4A	0.01511(17)	0.01513(14)	0.01128(13)	0.00076(11)	0.00604(12)	0.00275(10)
N5A	0.01274(17)	0.01731(15)	0.01338(14)	0.00377(12)	0.00146(12)	-0.00020(11)
N6A	0.01499(17)	0.01570(14)	0.01265(13)	0.00011(11)	0.00774(12)	0.00063(10)
C6A	0.01110(16)	0.01134(13)	0.00901(12)	0.00039(11)	0.00359(11)	0.00026(10)
C7A	0.01154(17)	0.01247(14)	0.00956(13)	0.00010(11)	0.00433(12)	0.00123(10)
C8A	0.01117(17)	0.01397(15)	0.01140(14)	0.00039(11)	0.00439(12)	0.00035(11)
C9A	0.01063(17)	0.01310(14)	0.01100(14)	0.00162(11)	0.00260(12)	0.00070(10)
C10A	0.01292(17)	0.01275(14)	0.00962(13)	0.00056(11)	0.00363(12)	0.00105(10)
C11A	0.01154(17)	0.01279(14)	0.00956(13)	-0.00008(11)	0.00469(12)	0.00035(10)
N1B	0.01333(16)	0.01406(14)	0.01295(14)	0.00148(11)	0.00415(12)	0.00146(10)
N3B	0.02002(19)	0.01569(15)	0.01234(14)	0.00281(13)	0.00577(13)	0.00248(11)
C2B	0.0163(2)	0.01362(15)	0.01608(17)	-0.00060(13)	0.00830(15)	-0.00008(12)
C4B	0.0167(2)	0.01852(18)	0.01645(17)	0.00137(14)	0.00926(15)	0.00208(14)
C5B	0.0147(2)	0.0204(2)	0.01844(19)	0.00349(15)	0.00588(16)	0.00110(15)
O1B	0.01247(15)	0.02135(16)	0.01652(14)	0.00495(11)	0.00582(12)	0.00550(11)
O2B	0.01587(17)	0.0348(2)	0.01282(14)	0.00266(14)	0.00319(12)	0.00644(13)
O3B	0.01728(18)	0.02724(19)	0.02597(19)	0.00160(14)	0.01258(15)	0.01028(15)
O4B	0.01460(18)	0.0272(2)	0.0307(2)	0.00688(14)	0.01031(16)	0.00731(16)
O5B	0.02145(19)	0.02520(18)	0.01980(17)	0.00795(14)	0.00555(14)	0.00887(13)
O6B	0.02485(19)	0.01831(15)	0.01902(15)	-0.00089(13)	0.01390(14)	0.00281(11)
O7B	0.0217(2)	0.0332(2)	0.0274(2)	0.01210(16)	0.01625(17)	0.00938(16)
N4B	0.01452(16)	0.01384(13)	0.01265(13)	0.00137(11)	0.00666(12)	0.00114(10)
N5B	0.01257(16)	0.01430(14)	0.01763(16)	0.00219(11)	0.00343(13)	0.00156(11)
N6B	0.01423(17)	0.01609(15)	0.01320(14)	-0.00104(11)	0.00704(12)	-0.00081(10)
C6B	0.01067(16)	0.01168(13)	0.01176(14)	0.00016(11)	0.00431(12)	0.00024(10)
C7B	0.01146(17)	0.01167(14)	0.01187(14)	0.00053(11)	0.00518(12)	0.00079(10)
C8B	0.01184(17)	0.01196(14)	0.01433(15)	0.00040(11)	0.00583(13)	0.00038(11)
C9B	0.01085(17)	0.01218(14)	0.01428(15)	0.00135(11)	0.00403(13)	0.00129(11)
C10B	0.01318(18)	0.01236(14)	0.01238(14)	0.00056(12)	0.00443(13)	0.00081(11)
C11B	0.01170(17)	0.01216(14)	0.01195(14)	-0.00027(11)	0.00553(12)	0.00020(10)

Table S11. Non-hydrogen anisotropic displacement parameters of the MM of BIPa, units = Å^2

	U11	U22	U33	U12	U13	U23
O8	0.0128(3)	0.0117(2)	0.0196(2)	-0.00044(17)	0.0054(2)	0.00023(17)
O9	0.0103(3)	0.0192(3)	0.0196(3)	-0.00024(19)	0.0068(2)	0.0034(2)
N7	0.01402(18)	0.01092(14)	0.01081(13)	0.00080(11)	0.00309(13)	-0.00014(10)
C12	0.00942(17)	0.01201(15)	0.01062(14)	-0.00072(12)	0.00316(12)	0.00069(11)
C13	0.00976(17)	0.01106(15)	0.01233(15)	-0.00008(11)	0.00365(13)	0.00122(11)
C14	0.0136(2)	0.01816(19)	0.01491(18)	0.00355(15)	0.00296(16)	0.00371(14)
C15	0.0181(2)	0.01387(19)	0.0263(3)	-0.00323(16)	0.0027(2)	0.00493(17)
C16	0.0446(4)	0.0213(2)	0.0170(2)	0.0055(3)	0.0152(3)	-0.00416(18)
N1A	0.01148(18)	0.01503(16)	0.01199(15)	0.00241(12)	0.00253(13)	0.00130(12)
N3A	0.01152(19)	0.01607(17)	0.01668(17)	0.00168(13)	0.00387(15)	-0.00049(13)
C2A	0.0149(2)	0.01480(17)	0.01598(18)	0.00144(14)	0.00699(16)	0.00259(14)
C4A	0.0156(2)	0.0239(2)	0.01112(17)	0.00543(17)	0.00239(16)	0.00174(15)
C5A	0.0150(2)	0.0241(2)	0.01269(17)	0.00390(17)	0.00593(16)	0.00237(15)
O1A	0.0123(2)	0.0173(2)	0.01180(19)	0.00585(18)	0.00434(18)	0.00362(16)
O2A	0.0170(3)	0.0344(4)	0.0135(2)	0.0065(3)	0.0064(2)	0.0111(2)
O3A	0.0209(3)	0.0228(3)	0.0183(3)	0.0032(3)	0.0132(3)	0.0074(2)
O4A	0.0167(4)	0.0371(5)	0.0227(4)	0.0137(3)	0.0080(3)	0.0041(3)
O5A	0.0192(3)	0.0227(3)	0.0167(3)	0.0073(2)	0.0022(2)	0.0072(2)
O6A	0.0226(4)	0.0368(4)	0.0123(2)	-0.0044(3)	0.0104(3)	-0.0010(2)
O7A	0.0127(3)	0.0305(4)	0.0194(3)	0.0027(2)	0.0094(3)	0.0050(3)
N4A	0.01374(19)	0.01497(15)	0.01022(13)	0.00095(12)	0.00553(13)	0.00334(11)
N5A	0.01178(18)	0.01683(17)	0.01252(15)	0.00445(13)	0.00165(13)	0.00054(12)
N6A	0.01344(19)	0.01590(16)	0.01146(14)	0.00033(12)	0.00710(13)	0.00070(11)
C6A	0.00976(16)	0.01074(14)	0.00850(13)	0.00121(11)	0.00353(12)	0.00089(10)
C7A	0.00996(17)	0.01163(14)	0.00892(13)	0.00054(11)	0.00405(12)	0.00122(10)
C8A	0.00964(17)	0.01344(16)	0.01062(14)	0.00112(12)	0.00415(13)	0.00097(11)
C9A	0.00984(18)	0.01262(15)	0.00969(14)	0.00161(12)	0.00261(13)	0.00096(11)
C10A	0.01152(18)	0.01244(15)	0.00867(13)	0.00118(12)	0.00333(12)	0.00181(11)
C11A	0.01022(17)	0.01180(14)	0.00840(13)	0.00075(11)	0.00400(12)	0.00106(10)
N1B	0.01227(18)	0.01325(15)	0.01230(14)	0.00170(12)	0.00391(13)	0.00192(11)
N3B	0.0195(2)	0.01482(16)	0.01143(15)	0.00328(14)	0.00571(15)	0.00299(12)
O1B	0.0111(3)	0.0217(3)	0.0161(2)	0.0056(2)	0.0063(2)	0.0068(2)
O2B	0.0145(3)	0.0345(4)	0.0125(2)	0.0019(3)	0.0039(2)	0.0072(2)
O3B	0.0173(3)	0.0256(3)	0.0235(3)	0.0024(3)	0.0113(3)	0.0105(3)
O4B	0.0140(3)	0.0264(4)	0.0295(4)	0.0080(3)	0.0099(3)	0.0087(3)
O5B	0.0199(4)	0.0247(3)	0.0196(3)	0.0089(3)	0.0058(3)	0.0087(3)
O6B	0.0239(3)	0.0177(3)	0.0189(3)	0.0002(2)	0.0144(3)	0.0029(2)
O7B	0.0224(4)	0.0314(4)	0.0263(4)	0.0118(3)	0.0171(3)	0.0097(3)
O1B	0.0111(3)	0.0217(3)	0.0161(2)	0.0056(2)	0.0063(2)	0.0068(2)
O2B	0.0145(3)	0.0345(4)	0.0125(2)	0.0019(3)	0.0039(2)	0.0072(2)
O3B	0.0173(3)	0.0256(3)	0.0235(3)	0.0024(3)	0.0113(3)	0.0105(3)
O4B	0.0140(3)	0.0264(4)	0.0295(4)	0.0080(3)	0.0099(3)	0.0087(3)
O5B	0.0199(4)	0.0247(3)	0.0196(3)	0.0089(3)	0.0058(3)	0.0087(3)
O6B	0.0239(3)	0.0177(3)	0.0189(3)	0.0002(2)	0.0144(3)	0.0029(2)
O7B	0.0224(4)	0.0314(4)	0.0263(4)	0.0118(3)	0.0171(3)	0.0097(3)
C2B	0.0149(2)	0.01301(16)	0.01527(17)	-0.00028(13)	0.00790(16)	0.00076(13)
C4B	0.0153(2)	0.01796(19)	0.01564(18)	0.00153(15)	0.00890(16)	0.00306(15)
C5B	0.0131(2)	0.0203(2)	0.01693(19)	0.00410(16)	0.00511(17)	0.00198(16)
C6B	0.00923(17)	0.01128(14)	0.01110(14)	0.00087(11)	0.00417(12)	0.00114(11)
C7B	0.00997(17)	0.01097(14)	0.01139(14)	0.00092(11)	0.00490(13)	0.00129(11)
C8B	0.01021(18)	0.01156(15)	0.01370(16)	0.00106(12)	0.00540(14)	0.00107(12)
C9B	0.01033(18)	0.01133(15)	0.01324(16)	0.00171(12)	0.00410(13)	0.00195(12)
C10B	0.01135(18)	0.01191(15)	0.01198(15)	0.00101(12)	0.00428(13)	0.00164(12)
C11B	0.01052(18)	0.01121(14)	0.01097(14)	0.00001(11)	0.00464(13)	0.00038(11)

Table S12. Hydrogen anisotropic displacement parameters obtained from SHADE of BIPa, units = Å^2

	U11	U22	U33	U12	U13	U23
H131	0.040787	0.03027	0.033358	0.002626	0.026543	0.000576
H132	0.021534	0.03048	0.031609	-0.00367	-0.004487	0.005556
H141	0.043051	0.026026	0.051305	0.013399	0.009673	0.006372
H142	0.042228	0.054338	0.021036	0.005453	0.011875	-0.00077
H143	0.024681	0.046948	0.0455	-0.004271	0.010312	0.013709
H151	0.034106	0.046419	0.048803	-0.013213	-0.007998	0.013075
H152	0.049523	0.044397	0.051425	-0.007212	0.031751	0.001975
H153	0.049052	0.020668	0.066732	0.003369	0.018673	0.008672
H161	0.066334	0.045327	0.047756	-0.004635	0.040395	-0.00692
H162	0.067116	0.053989	0.022966	0.002254	0.003045	-0.007358
H163	0.096903	0.025887	0.051247	0.016033	0.038039	-0.0027
H1A	0.021344	0.034622	0.026395	0.007845	0.002617	0.005924
H2A	0.041833	0.041304	0.031752	0.004268	0.02252	0.012703
H3A	0.018162	0.038154	0.04423	0.002831	0.012137	0.000268
H4A	0.036805	0.053809	0.019979	0.014531	0.003088	0.010759
H5A	0.03257	0.057958	0.036011	0.007125	0.023474	0.007602
H8A	0.022781	0.039449	0.029346	0.005375	0.01716	0.004159
H10A	0.032016	0.033248	0.019641	0.004824	0.010754	0.011393
H1B	0.020901	0.03169	0.024287	0.005926	0.006178	0.009555
H2B	0.023034	0.033284	0.032512	0.000048	0.016187	0.00475
H3B	0.031887	0.032525	0.022999	0.00627	0.010972	0.011693
H4B	0.030058	0.035333	0.029388	0.001986	0.017977	0.010391
H5B	0.018974	0.039798	0.033044	0.008558	0.008333	0.007312
H8B	0.021487	0.035974	0.03423	0.003714	0.017734	0.006451
H10B	0.032373	0.029315	0.022673	0.004664	0.012428	0.01035

Table S13. Non-hydrogen anisotropic displacement parameters of the HAR (HF/def2-SVP) of BIPa, units = Å^2

	U11	U22	U33	U12	U13	U23
O8	0.0133(1)	0.0120(1)	0.0194(1)	-0.00091(8)	0.0053(1)	0.00029(9)
O9	0.0109(1)	0.0193(1)	0.0197(1)	-0.00022(9)	0.0069(1)	0.0034(1)
N7	0.0146(1)	0.0116(1)	0.0110(1)	0.00092(9)	0.0031(1)	-0.00019(9)
C12	0.0100(1)	0.0124(1)	0.0113(1)	-0.00062(9)	0.0033(1)	0.0008(1)
C13	0.0103(1)	0.0118(1)	0.0129(2)	-0.00022(9)	0.0039(1)	0.0013(1)
C14	0.0145(2)	0.0184(2)	0.0154(2)	0.0034(1)	0.0035(1)	0.0037(1)
C15	0.0191(2)	0.0141(2)	0.0271(2)	-0.0029(1)	0.0032(2)	0.0049(1)
C16	0.0437(3)	0.0219(2)	0.0178(3)	0.0054(2)	0.0146(2)	-0.0042(2)
N1A	0.0122(1)	0.0154(1)	0.0122(1)	0.0023(1)	0.0025(1)	0.0012(1)
N3A	0.0120(1)	0.0160(1)	0.0174(2)	0.0016(1)	0.0040(1)	-0.0008(1)
C2A	0.0158(2)	0.0155(1)	0.0162(2)	0.0016(1)	0.0071(1)	0.0026(1)
C4A	0.0160(2)	0.0245(2)	0.0116(2)	0.0054(1)	0.0022(1)	0.0017(1)
C5A	0.0155(2)	0.0248(2)	0.0133(2)	0.0042(1)	0.0060(1)	0.0026(1)
O1A	0.0130(1)	0.0174(1)	0.0121(1)	0.00522(9)	0.0049(1)	0.00345(9)
O2A	0.0181(1)	0.0340(2)	0.0137(1)	0.0062(1)	0.0065(1)	0.0107(1)
O3A	0.0202(1)	0.0238(2)	0.0181(2)	0.0031(1)	0.0122(1)	0.0075(1)
O4A	0.0169(1)	0.0369(2)	0.0224(2)	0.0129(1)	0.0073(1)	0.0045(2)
O5A	0.0190(2)	0.0232(2)	0.0169(1)	0.0074(1)	0.0021(1)	0.0064(1)
O6A	0.0222(2)	0.0373(2)	0.0128(2)	-0.0042(1)	0.0103(1)	-0.0012(1)
O7A	0.0133(1)	0.0310(2)	0.0193(2)	0.0024(1)	0.0093(1)	0.0048(1)
N4A	0.0142(1)	0.0157(1)	0.0105(1)	0.00110(9)	0.0057(1)	0.0035(1)
N5A	0.0122(1)	0.0175(1)	0.0129(1)	0.0046(1)	0.0018(1)	0.0009(1)
N6A	0.0138(1)	0.0171(1)	0.0115(1)	0.00021(9)	0.0072(1)	0.0007(1)
C6A	0.0102(1)	0.0115(1)	0.0090(1)	0.00136(9)	0.0036(1)	0.0011(1)
C7A	0.0108(1)	0.0121(1)	0.0095(1)	0.00026(9)	0.0041(1)	0.0010(1)
C8A	0.0101(1)	0.0144(1)	0.0110(1)	0.0013(1)	0.0042(1)	0.0013(1)
C9A	0.0105(1)	0.0129(1)	0.0104(1)	0.00150(9)	0.0027(1)	0.0007(1)
C10A	0.0121(1)	0.0131(1)	0.0093(1)	0.0013(1)	0.0037(1)	0.0022(1)
C11A	0.0111(1)	0.0122(1)	0.0090(1)	0.00048(9)	0.0040(1)	0.0008(1)
N1B	0.0128(1)	0.0137(1)	0.0125(1)	0.00181(9)	0.0040(1)	0.0017(1)
N3B	0.0198(2)	0.0151(1)	0.0121(2)	0.0028(1)	0.0059(1)	0.0026(1)
C2B	0.0155(2)	0.0139(1)	0.0158(2)	-0.0001(1)	0.0082(1)	0.0008(1)
C4B	0.0159(2)	0.0184(2)	0.0161(2)	0.0014(1)	0.0089(1)	0.0031(1)
C5B	0.0138(2)	0.0206(2)	0.0174(2)	0.0041(1)	0.0052(1)	0.0017(1)
O1B	0.0123(1)	0.0216(1)	0.0160(1)	0.00489(9)	0.0065(1)	0.0062(1)
O2B	0.0159(1)	0.0340(2)	0.0128(1)	0.0022(1)	0.0044(1)	0.0071(1)
O3B	0.0170(1)	0.0270(2)	0.0239(2)	0.0028(1)	0.0114(1)	0.0114(1)
O4B	0.0139(1)	0.0267(2)	0.0293(2)	0.0077(1)	0.0092(1)	0.0087(1)
O5B	0.0198(2)	0.0249(2)	0.0202(2)	0.0088(1)	0.0057(1)	0.0085(1)
O6B	0.0236(1)	0.0185(1)	0.0190(2)	0.0000(1)	0.0143(1)	0.0023(1)
O7B	0.0223(1)	0.0326(2)	0.0269(2)	0.0114(1)	0.0174(1)	0.0103(2)
N4B	0.0131(1)	0.0144(1)	0.0123(1)	0.00131(9)	0.0064(1)	0.0021(1)
N5B	0.0119(1)	0.0145(1)	0.0173(2)	0.0031(1)	0.0037(1)	0.0027(1)
N6B	0.0134(1)	0.0159(1)	0.0137(2)	0.00002(9)	0.0076(1)	0.0002(1)
C6B	0.0098(1)	0.0120(1)	0.0117(1)	0.00087(9)	0.0044(1)	0.0012(1)
C7B	0.0107(1)	0.0112(1)	0.0120(1)	0.00071(9)	0.0050(1)	0.0010(1)
C8B	0.0107(1)	0.0124(1)	0.0143(2)	0.00123(9)	0.0057(1)	0.0016(1)
C9B	0.0109(1)	0.0118(1)	0.0138(2)	0.0014(1)	0.0041(1)	0.0015(1)
C10B	0.0118(1)	0.0129(1)	0.0125(2)	0.0011(1)	0.0047(1)	0.0019(1)
C11B	0.0111(1)	0.0119(1)	0.0114(1)	-0.00004(9)	0.0047(1)	0.0003(1)

Table S14. Hydrogen anisotropic displacement parameters of the HAR (HF/def2-SVP) of BIPa, units = Å^2

	U11	U22	U33	U12	U13	U23
H131	0.029(5)	0.026(5)	0.027(6)	-0.005(4)	0.010(4)	0.007(4)
H132	0.018(4)	0.013(4)	0.032(5)	-0.010(3)	0.000(4)	0.000(4)
H141	0.038(6)	0.048(7)	0.039(8)	-0.004(5)	0.012(5)	0.000(6)
H142	0.040(6)	0.050(7)	0.025(6)	-0.002(5)	0.008(5)	-0.008(5)
H143	0.031(5)	0.034(6)	0.024(6)	0.010(4)	0.007(4)	0.011(5)
H151	0.047(7)	0.016(5)	0.051(7)	-0.010(5)	-0.002(5)	0.006(5)
H152	0.048(6)	0.036(6)	0.041(8)	-0.005(4)	0.029(6)	0.002(6)
H153	0.043(7)	0.053(8)	0.051(8)	-0.006(6)	0.003(6)	-0.002(6)
H161	0.065(6)	0.061(9)	0.04(1)	0.038(6)	0.039(7)	0.006(7)
H162	0.082(8)	0.045(7)	0.032(9)	0.001(6)	0.025(7)	-0.015(6)
H163	0.12(1)	0.036(7)	0.04(1)	0.010(6)	0.046(9)	0.002(7)
H1A	0.028(6)	0.022(7)	0.042(9)	0.011(5)	0.021(6)	0.013(6)
H2A	0.046(7)	0.11(1)	0.024(7)	0.003(7)	0.008(6)	-0.008(7)
H3A	0.034(5)	0.022(5)	0.041(7)	0.008(4)	0.022(5)	0.003(5)
H4A	0.052(6)	0.054(8)	0.038(9)	0.028(5)	0.023(6)	0.016(6)
H5A	0.027(5)	0.032(6)	0.024(6)	0.008(4)	0.010(4)	0.004(5)
H8A	0.037(5)	0.026(5)	0.034(7)	0.006(4)	0.015(5)	0.005(5)
H10A	0.005(4)	0.022(6)	0.033(7)	-0.003(4)	0.010(4)	0.003(5)
H1B	0.030(5)	0.012(5)	0.037(9)	-0.015(4)	0.023(5)	-0.012(5)
H2B	0.055(5)	0.024(5)	0.045(9)	-0.015(4)	0.040(5)	-0.003(5)
H3B	0.073(8)	0.037(8)	0.030(9)	-0.007(6)	0.027(7)	-0.006(6)
H4B	0.048(5)	0.030(6)	0.034(7)	-0.008(4)	0.022(5)	0.005(5)
H5B	0.030(5)	0.054(8)	0.040(7)	0.011(5)	0.011(5)	0.002(6)
H8B	0.013(4)	0.021(5)	0.029(6)	0.000(3)	0.006(4)	0.003(4)
H10B	0.032(5)	0.018(5)	0.029(6)	0.004(4)	0.015(4)	0.008(4)

Table S15. Non-hydrogen anisotropic displacement parameters of the HAR (HF/def2-TZVP) of BIPa, units = Å^2

	U11	U22	U33	U12	U13	U23
O8	0.0132(1)	0.0118(1)	0.0194(1)	-0.00080(8)	0.0051(1)	0.00032(9)
O9	0.0129(1)	0.0173(1)	0.0120(1)	0.00532(9)	0.0048(1)	0.00343(9)
N7	0.0126(1)	0.0137(1)	0.0125(1)	0.00192(9)	0.0039(1)	0.0019(1)
C12	0.0103(1)	0.0129(1)	0.0104(1)	0.00153(9)	0.0028(1)	0.0008(1)
C13	0.0109(1)	0.0121(1)	0.0088(1)	0.00058(9)	0.0040(1)	0.0009(1)
C14	0.0119(1)	0.0130(1)	0.0093(1)	0.0014(1)	0.0036(1)	0.0023(1)
C15	0.0106(1)	0.0120(1)	0.0095(1)	0.00037(9)	0.0042(1)	0.0011(1)
C16	0.0105(1)	0.0111(1)	0.0120(1)	0.00069(9)	0.0050(1)	0.0010(1)
N1A	0.0128(1)	0.0144(1)	0.0121(1)	0.00130(9)	0.0063(1)	0.0021(1)
N3A	0.0135(1)	0.0171(1)	0.0114(1)	0.00013(9)	0.0072(1)	0.0006(1)
C2A	0.0110(1)	0.0117(1)	0.0113(1)	0.00005(9)	0.0047(1)	0.0003(1)
C4A	0.0096(1)	0.0119(1)	0.0115(1)	0.00092(9)	0.0043(1)	0.0012(1)
C5A	0.0100(1)	0.0114(1)	0.0088(1)	0.00141(9)	0.0035(1)	0.0011(1)
O1A	0.0108(1)	0.0192(1)	0.0198(1)	-0.00014(9)	0.0069(1)	0.0035(1)
O2A	0.0122(1)	0.0216(1)	0.0157(1)	0.00501(9)	0.0062(1)	0.0062(1)
O3A	0.0192(2)	0.0232(2)	0.0165(1)	0.0074(1)	0.0020(1)	0.0065(1)
O4A	0.0200(1)	0.0239(2)	0.0181(2)	0.0031(1)	0.0122(1)	0.0075(1)
O5A	0.0179(1)	0.0340(2)	0.0137(1)	0.0063(1)	0.0064(1)	0.0108(1)
O6A	0.0236(1)	0.0183(1)	0.0189(2)	-0.0000(1)	0.0142(1)	0.0025(1)
O7A	0.0168(1)	0.0270(2)	0.0241(2)	0.0027(1)	0.0115(1)	0.0113(1)
N4A	0.0121(1)	0.0155(1)	0.0120(1)	0.0023(1)	0.0025(1)	0.0012(1)
N5A	0.0139(1)	0.0156(1)	0.0103(1)	0.00118(9)	0.0056(1)	0.0036(1)
N6A	0.0118(1)	0.0160(1)	0.0173(2)	0.0016(1)	0.0039(1)	-0.0008(1)
C6A	0.0106(1)	0.0124(1)	0.0142(2)	0.00126(9)	0.0056(1)	0.0016(1)
C7A	0.0100(1)	0.0142(1)	0.0110(1)	0.0013(1)	0.0043(1)	0.0012(1)
C8A	0.0099(1)	0.0123(1)	0.0111(1)	-0.00067(9)	0.0032(1)	0.0006(1)
C9A	0.0153(2)	0.0138(1)	0.0157(2)	-0.0002(1)	0.0082(1)	0.0009(1)
C10A	0.0108(1)	0.0116(1)	0.0138(2)	0.0014(1)	0.0042(1)	0.0016(1)
C11A	0.0117(1)	0.0129(1)	0.0122(1)	0.0011(1)	0.0045(1)	0.0018(1)
N1B	0.0132(1)	0.0157(1)	0.0136(1)	0.00008(9)	0.0076(1)	0.0003(1)
N3B	0.0144(1)	0.0115(1)	0.0109(1)	0.00097(9)	0.0031(1)	-0.00016(9)
C2B	0.0157(2)	0.0155(1)	0.0160(2)	0.0016(1)	0.0071(1)	0.0025(1)
C4B	0.0102(1)	0.0118(1)	0.0127(2)	-0.00026(9)	0.0038(1)	0.0013(1)
C5B	0.0157(2)	0.0183(2)	0.0161(2)	0.0014(1)	0.0089(1)	0.0031(1)
O1B	0.0129(1)	0.0310(2)	0.0195(2)	0.0022(1)	0.0092(1)	0.0047(1)
O2B	0.0157(1)	0.0340(2)	0.0127(1)	0.0022(1)	0.0042(1)	0.0071(1)
O3B	0.0198(2)	0.0249(2)	0.0200(2)	0.0088(1)	0.0057(1)	0.0087(1)
O4B	0.0137(1)	0.0267(2)	0.0292(2)	0.0076(1)	0.0093(1)	0.0088(1)
O5B	0.0223(2)	0.0373(2)	0.0125(2)	-0.0041(1)	0.0102(1)	-0.0012(1)
O6B	0.0168(1)	0.0371(2)	0.0223(2)	0.0129(1)	0.0074(1)	0.0045(2)
O7B	0.0222(1)	0.0323(2)	0.0269(2)	0.0116(1)	0.0174(1)	0.0104(2)
N4B	0.0117(1)	0.0143(1)	0.0170(1)	0.0031(1)	0.0036(1)	0.0027(1)
N5B	0.0196(2)	0.0152(1)	0.0120(2)	0.0028(1)	0.0059(1)	0.0027(1)
N6B	0.0119(1)	0.0175(1)	0.0129(1)	0.0047(1)	0.0019(1)	0.0009(1)
C6B	0.0137(2)	0.0206(2)	0.0173(2)	0.0041(1)	0.0052(1)	0.0017(1)
C7B	0.0158(2)	0.0244(2)	0.0115(2)	0.0053(1)	0.0021(1)	0.0016(1)
C8B	0.0144(2)	0.0182(2)	0.0154(2)	0.0034(1)	0.0034(1)	0.0037(1)
C9B	0.0153(2)	0.0247(2)	0.0132(2)	0.0042(1)	0.0060(1)	0.0027(1)
C10B	0.0189(2)	0.0141(2)	0.0273(2)	-0.0029(1)	0.0032(2)	0.0051(1)
C11B	0.0435(3)	0.0218(2)	0.0176(3)	0.0052(2)	0.0144(2)	-0.0044(2)

Table S16. Hydrogen anisotropic displacement parameters of the HAR (HF/def2-TZVP) of BIPa, units = Å^2

	U11	U22	U33	U12	U13	U23
H131	0.033(5)	0.031(5)	0.024(6)	-0.001(4)	0.012(4)	0.003(4)
H132	0.020(4)	0.014(4)	0.035(5)	-0.009(3)	0.001(4)	-0.000(4)
H141	0.040(6)	0.049(7)	0.046(8)	0.003(5)	0.014(5)	-0.000(6)
H142	0.043(5)	0.049(7)	0.025(6)	-0.006(5)	0.013(5)	-0.007(5)
H143	0.033(5)	0.043(6)	0.035(7)	0.005(4)	0.012(5)	0.014(5)
H151	0.041(7)	0.023(5)	0.053(7)	-0.006(5)	-0.007(5)	0.013(5)
H152	0.050(5)	0.052(7)	0.036(8)	-0.003(5)	0.031(6)	0.002(6)
H153	0.050(7)	0.038(6)	0.060(8)	-0.002(5)	0.007(6)	0.002(6)
H161	0.060(6)	0.058(8)	0.05(1)	0.026(5)	0.040(6)	0.005(7)
H162	0.080(8)	0.055(8)	0.030(8)	-0.004(6)	0.023(7)	-0.019(6)
H163	0.12(1)	0.039(7)	0.04(1)	0.015(6)	0.049(9)	0.002(7)
H1A	0.035(6)	0.021(6)	0.041(9)	0.008(5)	0.021(6)	0.010(6)
H2A	0.061(8)	0.09(1)	0.022(7)	0.017(7)	0.005(6)	0.004(7)
H3A	0.036(5)	0.043(6)	0.035(7)	0.010(4)	0.023(5)	0.004(5)
H4A	0.048(6)	0.060(8)	0.043(8)	0.022(5)	0.026(6)	0.013(6)
H5A	0.028(5)	0.035(6)	0.026(6)	0.009(4)	0.012(4)	0.001(5)
H8A	0.042(5)	0.029(5)	0.032(7)	0.007(4)	0.015(5)	0.007(5)
H10A	0.004(4)	0.023(6)	0.028(6)	0.002(4)	0.003(4)	0.006(5)
H1B	0.035(5)	0.024(5)	0.024(6)	0.007(4)	0.014(4)	0.011(4)
H2B	0.054(5)	0.026(5)	0.046(9)	-0.015(4)	0.039(5)	-0.000(5)
H3B	0.072(8)	0.048(8)	0.03(1)	0.002(6)	0.029(7)	-0.000(7)
H4B	0.054(5)	0.031(5)	0.041(8)	-0.009(4)	0.030(5)	0.009(5)
H5B	0.028(5)	0.060(8)	0.042(7)	0.008(5)	0.012(5)	0.003(6)
H8B	0.010(4)	0.031(5)	0.030(6)	-0.002(3)	0.006(4)	0.001(4)
H10B	0.038(5)	0.016(5)	0.039(9)	-0.012(4)	0.022(6)	-0.010(5)

Table S17. Non-hydrogen anisotropic displacement parameters of the HAR (HF/def2-TZVP, charges) of BIPa, units = Å^2

	U11	U22	U33	U12	U13	U23
O8	0.0132(1)	0.0119(1)	0.0195(1)	-0.00079(8)	0.0052(1)	0.00034(9)
O9	0.0108(1)	0.0192(1)	0.0198(1)	-0.00017(9)	0.0070(1)	0.0035(1)
N7	0.0145(1)	0.0114(1)	0.0109(1)	0.00095(9)	0.0032(1)	-0.00016(9)
C12	0.0099(1)	0.0123(1)	0.0113(1)	-0.00056(9)	0.0033(1)	0.0008(1)
C13	0.0102(1)	0.0117(1)	0.0127(1)	-0.00021(9)	0.0037(1)	0.0014(1)
C14	0.0145(2)	0.0182(2)	0.0156(2)	0.0034(1)	0.0036(1)	0.0038(1)
C15	0.0190(2)	0.0140(2)	0.0270(2)	-0.0029(1)	0.0032(2)	0.0049(1)
C16	0.0439(3)	0.0216(2)	0.0178(3)	0.0054(2)	0.0147(2)	-0.0042(2)
N1A	0.0120(1)	0.0154(1)	0.0120(1)	0.0023(1)	0.0024(1)	0.0012(1)
N3A	0.0120(1)	0.0159(1)	0.0173(2)	0.0016(1)	0.0040(1)	-0.0007(1)
C2A	0.0156(2)	0.0156(1)	0.0159(2)	0.0016(1)	0.0069(1)	0.0026(1)
C4A	0.0157(2)	0.0245(2)	0.0115(2)	0.0054(1)	0.0020(1)	0.0017(1)
C5A	0.0154(2)	0.0246(2)	0.0132(2)	0.0042(1)	0.0061(1)	0.0026(1)
O1A	0.0128(1)	0.0173(1)	0.0120(1)	0.00535(9)	0.0048(1)	0.00352(9)
O2A	0.0179(1)	0.0340(2)	0.0135(1)	0.0063(1)	0.0062(1)	0.0108(1)
O3A	0.0200(1)	0.0239(2)	0.0182(2)	0.0031(1)	0.0123(1)	0.0074(1)
O4A	0.0168(1)	0.0371(2)	0.0224(2)	0.0129(1)	0.0075(1)	0.0046(2)
O5A	0.0192(2)	0.0231(2)	0.0168(1)	0.0074(1)	0.0022(1)	0.0065(1)
O6A	0.0223(2)	0.0373(2)	0.0126(2)	-0.0042(1)	0.0103(1)	-0.0012(1)
O7A	0.0130(1)	0.0311(2)	0.0193(2)	0.0023(1)	0.0091(1)	0.0049(1)
N4A	0.0139(1)	0.0156(1)	0.0104(1)	0.00121(9)	0.0057(1)	0.0036(1)
N5A	0.0121(1)	0.0174(1)	0.0128(1)	0.0047(1)	0.0020(1)	0.0009(1)
N6A	0.0135(1)	0.0171(1)	0.0113(1)	0.00016(9)	0.0071(1)	0.0007(1)
C6A	0.0100(1)	0.0114(1)	0.0089(1)	0.00142(9)	0.0036(1)	0.0011(1)
C7A	0.0105(1)	0.0121(1)	0.0094(1)	0.00037(9)	0.0041(1)	0.0010(1)
C8A	0.0100(1)	0.0143(1)	0.0109(1)	0.0013(1)	0.0042(1)	0.0013(1)
C9A	0.0103(1)	0.0129(1)	0.0102(1)	0.00150(9)	0.0026(1)	0.0008(1)
C10A	0.0120(1)	0.0130(1)	0.0093(1)	0.0014(1)	0.0037(1)	0.0022(1)
C11A	0.0109(1)	0.0121(1)	0.0088(1)	0.00059(9)	0.0040(1)	0.0009(1)
N1B	0.0126(1)	0.0137(1)	0.0123(1)	0.00180(9)	0.0038(1)	0.0018(1)
N3B	0.0197(2)	0.0151(1)	0.0120(2)	0.0028(1)	0.0060(1)	0.0027(1)
C2B	0.0151(2)	0.0139(1)	0.0156(2)	-0.0001(1)	0.0080(1)	0.0009(1)
C4B	0.0158(2)	0.0182(2)	0.0162(2)	0.0015(1)	0.0090(1)	0.0033(1)
C5B	0.0136(2)	0.0206(2)	0.0172(2)	0.0041(1)	0.0051(1)	0.0018(1)
O1B	0.0122(1)	0.0217(1)	0.0159(1)	0.00503(9)	0.0063(1)	0.0063(1)
O2B	0.0158(1)	0.0340(2)	0.0127(1)	0.0023(1)	0.0043(1)	0.0071(1)
O3B	0.0168(1)	0.0270(2)	0.0240(2)	0.0027(1)	0.0114(1)	0.0114(1)
O4B	0.0136(1)	0.0268(2)	0.0292(2)	0.0076(1)	0.0092(1)	0.0087(1)
O5B	0.0199(2)	0.0249(2)	0.0200(2)	0.0088(1)	0.0058(1)	0.0086(1)
O6B	0.0237(1)	0.0184(1)	0.0190(2)	0.0000(1)	0.0143(1)	0.0025(1)
O7B	0.0221(1)	0.0323(2)	0.0269(2)	0.0115(1)	0.0173(1)	0.0103(2)
N4B	0.0127(1)	0.0144(1)	0.0121(1)	0.00134(9)	0.0063(1)	0.0021(1)
N5B	0.0118(1)	0.0144(1)	0.0171(1)	0.0032(1)	0.0037(1)	0.0028(1)
N6B	0.0132(1)	0.0157(1)	0.0135(1)	0.00012(9)	0.0076(1)	0.0003(1)
C6B	0.0096(1)	0.0119(1)	0.0115(1)	0.00091(9)	0.0043(1)	0.0012(1)
C7B	0.0104(1)	0.0112(1)	0.0119(1)	0.00076(9)	0.0049(1)	0.0010(1)
C8B	0.0105(1)	0.0124(1)	0.0140(2)	0.00123(9)	0.0054(1)	0.0016(1)
C9B	0.0106(1)	0.0118(1)	0.0135(2)	0.00138(9)	0.0040(1)	0.0016(1)
C10B	0.0117(1)	0.0128(1)	0.0124(2)	0.0012(1)	0.0046(1)	0.0019(1)
C11B	0.0109(1)	0.0118(1)	0.0113(1)	0.00007(9)	0.0046(1)	0.0003(1)

Table S18. Hydrogen anisotropic displacement parameters of the HAR (HF/def2-TZVP, charges) of BIPa, units = Å^2

	U11	U22	U33	U12	U13	U23
H131	0.036(5)	0.030(5)	0.028(6)	-0.001(4)	0.016(4)	0.003(4)
H132	0.021(4)	0.013(4)	0.035(5)	-0.009(3)	0.001(4)	0.001(4)
H141	0.043(6)	0.049(7)	0.044(8)	0.004(5)	0.015(5)	0.000(6)
H142	0.041(5)	0.051(7)	0.023(6)	-0.006(5)	0.010(5)	-0.008(5)
H143	0.033(5)	0.045(6)	0.027(6)	0.004(4)	0.007(4)	0.013(5)
H151	0.043(7)	0.023(5)	0.052(7)	-0.008(5)	-0.006(5)	0.011(5)
H152	0.050(5)	0.049(7)	0.038(8)	-0.002(5)	0.031(5)	0.004(6)
H153	0.052(7)	0.036(6)	0.064(8)	-0.004(5)	0.010(6)	0.002(6)
H161	0.061(6)	0.057(8)	0.05(1)	0.025(5)	0.041(6)	0.004(7)
H162	0.079(8)	0.052(8)	0.032(9)	-0.001(6)	0.024(7)	-0.014(6)
H163	0.12(1)	0.039(7)	0.04(1)	0.016(6)	0.052(9)	0.002(7)
H1A	0.002(4)	0.026(6)	0.027(6)	0.001(4)	0.000(4)	0.007(5)
H2A	0.036(5)	0.040(6)	0.036(7)	0.011(4)	0.023(5)	0.004(5)
H3A	0.033(6)	0.021(6)	0.046(9)	0.007(4)	0.024(6)	0.007(6)
H4A	0.056(8)	0.09(1)	0.026(7)	0.011(6)	0.005(6)	-0.002(7)
H5A	0.049(6)	0.059(8)	0.042(8)	0.023(5)	0.026(6)	0.015(6)
H8A	0.028(4)	0.034(6)	0.029(6)	0.010(4)	0.014(4)	0.004(5)
H10A	0.045(5)	0.028(5)	0.036(7)	0.008(4)	0.020(5)	0.009(5)
H1B	0.033(5)	0.019(6)	0.032(8)	-0.010(4)	0.016(5)	-0.006(5)
H2B	0.056(5)	0.026(5)	0.050(9)	-0.015(4)	0.042(5)	-0.002(5)
H3B	0.075(8)	0.047(8)	0.029(9)	0.001(6)	0.028(7)	0.000(7)
H4B	0.051(5)	0.031(5)	0.040(7)	-0.008(4)	0.026(5)	0.009(5)
H5B	0.025(5)	0.063(8)	0.038(7)	0.010(5)	0.008(5)	0.005(6)
H8B	0.011(4)	0.032(5)	0.033(6)	-0.001(3)	0.010(4)	0.002(4)
H10B	0.038(5)	0.021(5)	0.030(6)	0.006(4)	0.019(4)	0.011(4)

Table S19. Anisotropic displacement parameters of the neutron data of KHOx, units = Å^2

	U11	U22	U33	U12	U13	U23
K1	0.0060(6)	0.0051(6)	0.0052(6)	0.0002(5)	0.0000(4)	-0.0007(5)
O1	0.0060(4)	0.0052(4)	0.0068(4)	0.0001(3)	-0.0006(3)	-0.0006(3)
O2	0.0087(4)	0.0054(4)	0.0077(4)	0.0012(3)	-0.0026(3)	0.0005(3)
O3	0.0055(4)	0.0066(4)	0.0073(4)	0.0014(3)	-0.0016(3)	-0.0004(3)
O4	0.0072(4)	0.005(4)	0.0067(4)	0.0000(3)	-0.0006(3)	-0.0008(3)
C1	0.0048(3)	0.0055(4)	0.0048(3)	0.0007(3)	-0.0005(3)	-0.0001(3)
C2	0.0042(3)	0.0047(4)	0.0051(3)	0.0001(3)	0.0000(3)	-0.0001(3)
H1	0.0169(9)	0.0161(9)	0.0169(9)	-0.0015(7)	0.0008(7)	-0.0017(7)

Table S20. Anisotropic displacement parameters of the IAM of KHOx, units = Å^2

	U11	U22	U33	U12	U13	U23
K1	0.00578(3)	0.00449(2)	0.00541(3)	0.00015(2)	-0.00008(2)	0.00007(2)
O1	0.00617(9)	0.00432(8)	0.00669(9)	0.00086(7)	-0.00108(7)	0.00002(7)
O2	0.00868(10)	0.00507(9)	0.00791(10)	-0.00037(7)	-0.00250(8)	-0.00150(7)
O3	0.00614(9)	0.00529(8)	0.00751(9)	0.00016(7)	-0.00148(7)	-0.00143(7)
O4	0.00721(9)	0.00450(8)	0.00667(9)	0.00128(7)	-0.00009(7)	0.00023(7)
C1	0.00478(10)	0.00467(10)	0.00483(10)	0.00016(8)	-0.00014(8)	-0.00027(8)
C2	0.00446(9)	0.00451(10)	0.00463(10)	-0.00006(8)	-0.00025(8)	-0.00002(8)

Table S21. Anisotropic displacement parameters of the MM of KHOx (hydrogen ADPs are obtained from SHADE), units = Å^2

	U11	U22	U33	U12	U13	U23
K1	0.00575(2)	0.004463(19)	0.00537(2)	-0.000075(16)	-0.000076(14)	-0.000139(15)
O1	0.00630(9)	0.00447(9)	0.00681(10)	0.00013(8)	-0.00102(8)	-0.00070(8)
O2	0.00878(11)	0.00514(9)	0.00807(10)	0.00147(8)	-0.00284(8)	0.00018(8)
O3	0.00637(9)	0.00536(9)	0.00759(10)	0.00137(8)	-0.00159(8)	-0.00037(8)
O4	0.00734(9)	0.00466(9)	0.00672(9)	-0.00008(8)	-0.00021(7)	-0.00136(8)
C1	0.00510(9)	0.00464(9)	0.00514(9)	0.00043(7)	-0.00044(7)	-0.00009(7)
C2	0.00474(8)	0.00437(8)	0.00501(9)	0.00026(7)	-0.00038(7)	-0.00005(7)
H1	0.1567	0.15272	0.015607	-0.004963	-0.000048	-0.004397

Table S22. Anisotropic displacement parameters of the HAR (HF/def2-SVP) of KHOx, units = Å^2

	U11	U22	U33	U12	U13	U23
K1	0.00539(2)	0.00411(2)	0.00500(2)	-0.00007(2)	-0.00012(2)	-0.00014(2)
O1	0.00645(8)	0.00455(7)	0.00689(8)	0.00028(6)	-0.00050(6)	-0.00060(6)
O2	0.00890(9)	0.00492(8)	0.00809(9)	0.00127(7)	-0.00319(7)	-0.00005(7)
O3	0.00625(8)	0.00552(8)	0.00745(8)	0.00112(7)	-0.00179(6)	-0.00055(7)
O4	0.00702(8)	0.00477(8)	0.00680(8)	0.00002(7)	-0.00060(6)	-0.00116(6)
C1	0.00497(9)	0.00471(9)	0.0051(1)	0.00043(7)	-0.00079(7)	-0.00002(7)
C2	0.00480(9)	0.00420(9)	0.00507(9)	0.00026(8)	-0.00047(7)	-0.00020(7)
H1	0.042(9)	0.15(2)	0.008(7)	0.06(1)	0.013(6)	-0.01(1)

Table S23. Anisotropic displacement parameters of the HAR (HF/def2-TZVP) of KHOx, units = Å^2

	U11	U22	U33	U12	U13	U23
K1	0.00537(2)	0.00410(2)	0.00498(2)	-0.00007(2)	-0.00012(2)	-0.00014(2)
O1	0.00643(8)	0.00450(8)	0.00688(8)	0.00026(6)	-0.00056(7)	-0.00063(6)
O2	0.00890(9)	0.00490(8)	0.00810(9)	0.00134(7)	-0.00320(7)	-0.00001(7)
O3	0.00624(8)	0.00548(8)	0.00751(9)	0.00118(7)	-0.00182(7)	-0.00053(7)
O4	0.00704(8)	0.00472(8)	0.00681(8)	0.00001(7)	-0.00057(6)	-0.00118(7)
C1	0.00498(9)	0.00464(9)	0.0051(1)	0.00045(8)	-0.00079(8)	-0.00000(7)
C2	0.00481(9)	0.00416(9)	0.0051(1)	0.00029(8)	-0.00048(7)	-0.00022(8)
H1	0.035(8)	0.14(2)	0.011(7)	0.05(1)	0.010(6)	-0.011(9)

Table S24. Anisotropic displacement parameters of the HAR (HF/def2-TZVP, cluster) of KHOx, units = Å^2

	U11	U22	U33	U12	U13	U23
K1	0.00537(2)	0.00410(2)	0.00501(2)	-0.00007(2)	-0.00013(2)	-0.00014(2)
O1	0.00620(8)	0.00464(8)	0.00684(9)	0.00024(7)	-0.00090(7)	-0.00065(7)
O2	0.0088(1)	0.00503(8)	0.00807(9)	0.00126(7)	-0.00292(7)	0.00007(7)
O3	0.00628(8)	0.00537(8)	0.00756(9)	0.00124(7)	-0.00192(7)	-0.00058(7)
O4	0.00708(8)	0.00485(8)	0.00672(9)	-0.00003(7)	-0.00040(7)	-0.00108(7)
C1	0.00501(9)	0.0045(1)	0.0051(1)	0.00037(8)	-0.00079(8)	-0.00012(8)
C2	0.00471(9)	0.00427(9)	0.0050(1)	0.00028(8)	-0.00048(7)	-0.00013(8)
H1	0.011(6)	0.025(7)	0.005(6)	0.003(6)	-0.001(5)	-0.004(5)

4 Hirshfeld rigid bond tests

Table S25. Averages of differences of mean square displacements amplitudes (DMSDA) in Å^2 along different kinds of bonds. The values in brackets are sample standard deviations obtained through averaging, not standard uncertainties. The tests are not applicable to the network compound KHOx.

				HAR		
bond type	Neutron	IAM	MM/SHADE	RHF/def2-SVP	RHF/def2-TZVP	RHF/def2-TZVP charges
			Rubrene			
C-C	0.000177(198)	0.000175(145)	0.000172(132)	0.000186(180)	0.000182(177)	0.000176(173)
C-H	0.0055(3)	–	0.0145(108)	0.0151(51)	0.0139(51)	0.0139(53)
			BIPa			
O-C	0.000300(216)	0.000918(346)	0.000275(171)	0.000410(195)	0.000355(161)	0.000370(226)
O-N	0.000442(334)	0.000933(299)	0.000633(505)	0.000937(378)	0.000874(390)	0.000936(372)
N-C	0.000233(175)	0.000478(278)	0.000261(220)	0.000596(523)	0.000527(337)	0.000531(339)
C-C	0.000393(209)	0.000280(211)	0.000207(153)	0.000478(630)	0.000257(148)	0.000274(169)
C-H	0.0053(11)	–	0.0048(9)	0.0201(119)	0.0154(80)	0.0155(82)
N-H	0.0063(11)	–	0.0053(2)	0.0189(136)	0.0200(147)	0.0170(106)
non-hydrogen	0.000339(245)	0.000565(375)	0.000337(338)	0.000635(521)	0.000516(372)	0.000538(384)
